# Cellular and Molecular Characterization of Multipolar Map5-Expressing Cells: A Subset of Newly Generated, Stage-Specific Parenchymal Cells in the Mammalian Central Nervous System

**DOI:** 10.1371/journal.pone.0063258

**Published:** 2013-05-07

**Authors:** Paola Crociara, Roberta Parolisi, Daniele Conte, Marta Fumagalli, Luca Bonfanti

**Affiliations:** 1 Neuroscience Institute Cavalieri Ottolenghi and Department of Veterinary Sciences, University of Turin, Turin, Italy; 2 Molecular Biotechnology Center, University of Turin, Turin, Italy; 3 Department of Pharmacological Sciences, University of Milan, Milan, Italy; Universidade Federal do ABC, Brazil

## Abstract

Although extremely interesting in adult neuro-glio-genesis and promising as an endogenous source for repair, parenchymal progenitors remain largely obscure in their identity and physiology, due to a scarce availability of stage-specific markers. What appears difficult is the distinction between real cell populations and various differentiation stages of the same population. Here we focused on a subset of multipolar, polydendrocyte-like cells (mMap5 cells) expressing the microtubule associated protein 5 (Map5), which is known to be present in most neurons. We characterized the morphology, phenotype, regional distribution, proliferative dynamics, and stage-specific marker expression of these cells in the rabbit and mouse CNS, also assessing their existence in other mammalian species. mMap5 cells were never found to co-express the Ng2 antigen. They appear to be a population of glial cells sharing features but also differences with Ng2+progenitor cells. We show that mMap5 cells are newly generated, postmitotic parenchymal elements of the oligodendroglial lineage, thus being a stage-specific population of polydendrocytes. Finally, we report that the number of mMap5 cells, although reduced within the brain of adult/old animals, can increase in neurodegenerative and traumatic conditions.

## Introduction

Parenchymal progenitors have become a hot research topic in neural plasticity since they represent intriguing players in adult neuro-glio-genesis and a promising source of endogenous elements for repair [1], [2], [3]. Most of them display neural developmental markers of the glial lineage, in the postnatal and adult central nervous system (CNS) being committed to the oligodendrocyte lineage and expressing a chondroitin sulfate proteoglycan (Nerve/glial antigen 2, Ng2; referred to as Ng2+cells [1], [4], [5]). The Ng2+cells are generally considered as synantocytes [6] or polydendrocytes [5], endowed with multiple functions in physiology and pathology which are still far from being utterly elucidated. A proportion of these cells persist in the adult CNS in a phenotypically immature form [1], [5], [7], most of which do continue to proliferate throughout life, thus being considered the main cycling population of the mature mammalian CNS [8]. Although parenchymal progenitors physiologically produce mainly glial cells [2], in some mammals/regions they can undergo spontaneous neurogenesis, e.g., in the rabbit striatum [9] and cerebellum [10]. Yet, also in the case of neuronal-committed cells, the primary progenitors remain poorly identified, in contrast with their progeny which is far more visible and characterized in its phenotype [9], [10]. The strong interest in better understanding parenchymal progenitors crashes against the many aspects which remain obscure about their identity, real nature, and physiology. Among these problems, a scarce availability of stage-specific markers along with a high heterogeneity linked to different variables (species, age, anatomical region, etc.), make the identification of subpopulations a hard task. More sneakily, what appears difficult is the distinction between real cell populations and various differentiation stages of the same population.

We have recently described a subset of glial-like cells immunoreactive for the microtubule associated protein 5 (Map5) in the rabbit cerebellum [10]. These cells show a morphology (ramified, multipolar) and a molecular signature (e.g., Olig2 expression) reminiscent of synantocytes/polydendrocytes, and some of them are newly generated within the mature cerebellar parenchyma [10]. Intriguingly, they express a cytoskeletal-associated molecule which is typically found in neurons (see Table 1). The Map5 molecule [33], also referred to as Map-1B [17], Map1X [34], or Map1.2 [35], belongs to a family of large and fibrous microtubule associated proteins (Maps) and shows a very wide range of expression in the CNS (summarized in Table 1). Map5 is the first Map detectable in neurons of the developing nervous system [36], [37], expressed at high levels in growing axons/growth cones and usually downregulated after cessation of axonal growth [25], [38] (reviewed in [39], [40]). Nevertheless, the protein remains expressed in the whole CNS during adulthood, its phosphorylated form reaching high levels within some regions endowed with plasticity [11], [12], [19], or under conditions that elicit axonal/synaptic plasticity in relation to physiological conditions and in response to injury [11], [41], . Map5 has also been implicated in a number of neurological disorders, such as fragile X syndrome [44], [45], giant axonal neuropathy [46] and Alzheimer disease [47], [48].

**Table 1:** Distribution of Map5 in the mammalian CNS as described in literature.

Species/region/method	Age	Cell type/distribution	Function	Refs
**Neuronal**				
Rat CNS	P0–P20, adult	most neurons (complementary to Map1A)	developmental/adult plasticity	[11]
	P3–P25, adult	most neurons	adult plasticity	[12]
Rat CNS and PNS	2,5 months	most neurons	plasticity, regeneration	[13]
Rat PNS	E18–P30, adult	peripheral nerves	axonal growth in nerve regeneration	[14]
Rat brain	3–24 months	most neurons		[15]
	adult	synaptic localization	synaptic plasticity	[16]
Rat brain and cultures	newborn	most neurons, some glial cells		[17]
Rat spinal cord	E9-postnatal	neuroblasts	morphogenic events	[18]
Rat olfactory bulb	adult	olfactory nerve, mitral and periglomerular cells	neurite outgrowth	[19]
Mouse brain	P5–P90	barrel cortex neurons	Plasticity	[20]
Mouse brain and cultures	E16	neurons, neuroblasts		[21]
Cat brain	P3–P28, adult	most neurons	axon/dendritic growth, microtubules stabilization	[22]
Human brain	13GW-17 years	most neurons	axonal growth	[23]
	18–33GW	ganglionic eminence, axonal bundles	axonal growth	[24]
Rat cell cultures	newborn	symphatethic neurons	axon extension	[25]
Mouse Map1b mutant cell cultures	E18–19	hippocampal pyramidal neurons	axon formation	[26]
**Glial**				
Rat brain and cultures	P7-adult	immature oligodendrocytes	myelination	[27]
Primary glial cultures		oligodendrocytes		[28]
		oligodendrocytes and type1 astrocytes		[29]
		oligodendrocytes, (negligible in astrocytes)	Formation/stabilization of myelin-forming processes	[30], [31]
CG4 cell line		oligodendrocytes		[32]

Map5 expression is not restricted to neuronal populations [27], [28], [29], [30], [32]. Only limited, heterogeneous information is available concerning its localization in other cell types (Table 1). It has mainly been reported in oligodendrocytes and Schwann cells that produce myelin in the central and peripheral nervous system [28], [41]. In particular, it is elevated in oligodendrocytes that initiate ensheathment of axons in the normal brain [27], and in Schwann cells during development and nerve regeneration [41]. Map5 is generally absent in astrocytes, although its expression in some subtypes, e.g., type1 astrocytes [29], is still controversial. Since most of these studies were carried out in culture [28], [29], [30], [31], [32], very little is known about the glial localization of Map5 in vivo.

Here we focused on the distribution of Map5 in the CNS of different mammalian species, with particular reference to a population of multipolar, glial-like cells (here referred to as mMap5 cells) in young/adult mice and rabbits. We characterized their morphology, phenotype, regional distribution, and stage-specific marker expression, as well as their occurrence in other mammals. Finally, we investigated the behavior of mMap5 cells in traumatic and neurodegenerative injury conditions in mice.

## Results

### Distribution of microtubule associated protein 5 (Map5) in neuronal and glial cell populations of the rabbit and mouse CNS

**Figure 1:**
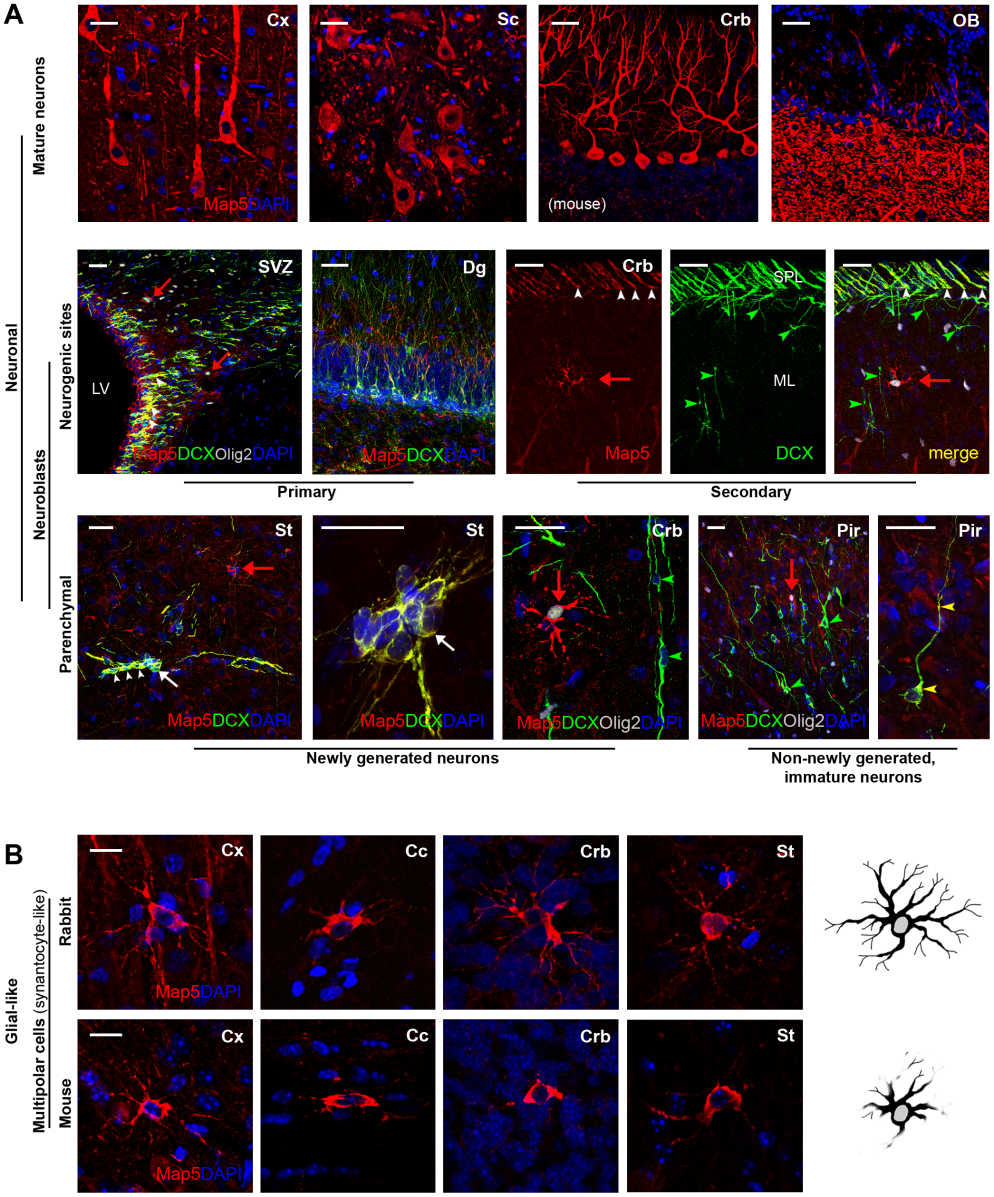
Map5 distribution in neuronal and glial cells of the rabbit and mouse CNS. A, Map5 is abundant in most populations of mature neurons (top), neuroblasts occurring in germinal layer-derived neurogenic sites or transitory germinative zones (e.g., rabbit subpial layer, SPL; middle), and in neural progenitors of the brain parenchyma (e.g., rabbit striatum, St; bottom, left). White arrows: clusters of newly generated neuroblasts; white arrowheads: chains of neuroblasts. Newly generated neurons in the cerebellum (Crb) and immature neurons of the piriform cortex (Pir) are generally Map5-negative (green arrowheads; bottom, right), apart from some immature neurons which show low level of Map5 staining (yellow arrowheads). Micrograph in panel A are from peripuberal and adult rabbit tissue, except those marked for mouse. For the Map5 staining on the SVZ ependymal wall, see Fig. S4B. B, In addition to its neuronal localization, Map5 decorates a population of multipolar cells with the morphology of synantocytes (see also red arrows in panel A). These cells are better visualized in rabbit than in mouse by immunocytochemistry, since staining in mice reveals to a lesser extent the ramifications of cell processes (schematically showed by drawings on the right; total length of cell processes quantifications in the two mammalian species is showed in Fig. S2A). Crb, cerebellum; ML, molecular layer; Cx, cerebral cortex; Cc, corpus callosum; SVZ, subventricular zone; LV, lateral ventricle; Sc, spinal cord; Dg, dentate gyrus of the hippocampus. Scale bars: A, 30 µm; B, 10 µm.

Firstly, we tested the cellular and regional distribution of Map5 in mature neurons of the rabbit and mouse CNS to check if the staining obtained with our antibodies was consistent with previous reports. The pattern of expression of Map5 in vivo, was analyzed at three different ages (3.5, 12, and 36 months) in rabbits, and two (40 days and 3 months) in mice, corresponding to peri-puberal and adult ages. Consistently with previous reports, Map5 was largely present in most CNS neurons (see references in Table 1 and Fig. 1A). This neuronal staining was detectable in apical dendrites and cell bodies but the nuclei were immunonegative. Large projection neurons, such as cerebral cortex pyramidal neurons, cerebellar Purkinje neurons, and spinal cord motorneurons were heavily immunostained for Map5 (Fig. 1A, top). As previously described in rodents [11], [16], cerebral cortex layers II, III, V were more intensely labeled (not shown) and staining in the olfactory bulb was restricted to the olfactory nerve and external plexiform layers (Fig. S1). No qualitative differences in the amount and distribution of Map5 in neurons were detectable at the different ages explored (see below for further age-related features).

In the whole CNS, the polyclonal anti-Map5 antiserum employed in our study and recognizing both unphosphorilated and phosphorilated isoforms, can be used interchangeably on rabbit and mouse tissues, yielding an identical staining pattern (Fig. S1). Once assessed the reliability of staining in mature neurons, we observed that Map5 is strongly expressed in several populations of neuroblasts which persist in the postnatal and adult brain (Fig. 1A, middle and bottom). They include newly generated neuronal precursors of germinal layer-derived neurogenic sites (the forebrain subventricular zone, SVZ, and the dentate gyrus of the hippocampus), neurogenic parenchymal progenitors of the rabbit striatum [9], and chain of neuroblasts in the transitory subpial layer (SPL), a secondary germinative zone of the postnatal rabbit cerebellum [49] (Fig. 1A). By contrast, Map5 is not present in immature or differentiating neurons, such as the PSA-NCAM+/DCX+ immature neurons in the piriform cortex (with rare exceptions; see Fig 1A, bottom) and the PSA-NCAM+/DCX+ newly generated neurons in the rabbit cerebellum (green arrowheads in Fig. 1A).

Beside its wide distribution in neuronal cells, Map5 is also detectable in a population of multipolar, glial-like cells widespread in most CNS grey and white matter (here referred to as mMap5 cells in order to distinguish them from the Map5+neuronal elements; Fig. 1B and red arrows in panel A). These cells, whose shape is reminiscent of parenchymal progenitors known as synantocytes/polydendrocytes [5], 6, were far more visible in the rabbit than in mouse, in the latter the ramified, multipolar processes appearing not completely stained (Fig. 1B). In order to assess this difference we quantified the total length of mMap5 cell processes in the grey (cerebral and cerebellar cortex) and white matter (corpus callosum) of the two species (Fig. S2A). Results indicated that rabbit mMap5 cell process ramifications are longer in both grey matter regions analyzed (cerebral cortex: rabbit 285,7±23,5 µm versus mouse 182,1±15,6 µm, P<0,05; cerebellar cortex: rabbit 485,9±29,5 µm versus mouse 127,1±12,3 µm, P<0,005), whereas they are similar within the corpus callosum (rabbit 93,7±0,5 µm versus mouse 57,0±13,5 µm, P>0,05). Such difference was more prominent in the cerebellar cortex with respect to cerebral cortex.

### Morphologic and phenotypic characterization of mMap5 cells

**Figure 2:**
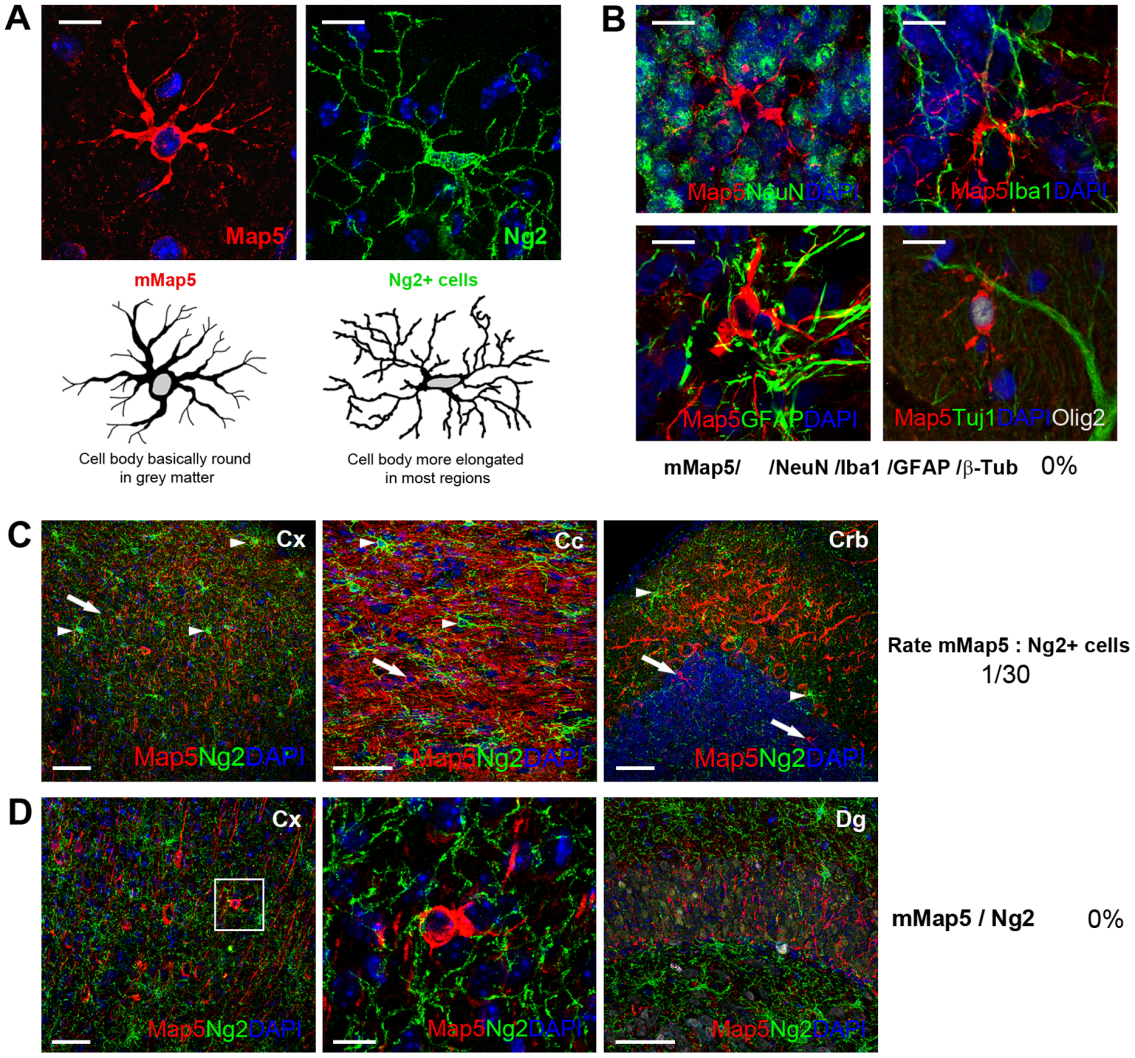
Morphological and phenotypic characterization of mMap5 cell population. A, Despite a substantially similar appearance, mMap5 cells are slightly different from Ng2+cells as to the shape of their cell body (quantifications of cell soma diameters is showed in Fig. S2A). The photographs are representative of two extremes: round-shaped and elongated cell bodies. B, Double staining with neuronal and glial antigens indicate that mMap5 cells are not neurons nor astrocytes or microglia. C, mMap5 cells (arrows) are ramified elements with a morphological appearance similar to Ng2+cells (arrowheads) but far less numerous. D, mMap5 cells and Ng2+cells belong to two distinct, non overlapping populations. Crb, cerebellum; Cx, cerebral cortex; Cc, corpus callosum; Dg, dentate gyrus of the hippocampus. Scale bars: A,B, 10 µm; C,D, 50 µm; inset in D, 10 µm.

In immunocytochemical specimens, multipolar Map5+cells (mMap5 cells) and Ng2+cells showed a similar, yet not identical, morphology (Fig. 2A). Processes revealed with Map5 are quite smooth, without the beads, swelling and varicosities that characterize the fine processes of Ng2+cells [1] (see Fig. 2A and Discussion). Since observations carried out on mMap5 cells in different CNS regions revealed various shapes, we performed a quantitative analysis of cell somata diameters, by measuring their minimum (min) and maximum extent (max) in mMap5 cells and Ng2+cells, within three brain regions (cerebral cortex, cerebellar cortex and corpus callosum; Fig. S2B). Comparison of the data obtained revealed the following trend: mMap5 cell somata were more frequently round-shaped in the cerebellar cortex (min 8,3±0,6 µm; max 9,4±0,5 µm; P>0,05), and, to a lesser extent, in the cerebral cortex (min 7,9±0,8 µm; max 10,3±0,8 µm; P<0,001), whereas Ng2 cell somata were more elongated (cerebral cortex: min 5,8±1,0 µm; max 10,4±1,2 µm; cerebellar cortex: min 5,9±0,9 µm; max 10,8±1,2 µm; P<0,001 in both regions). On the other hand, somata of both cell types were prevalently elongated in the white matter of the corpus callosum (mMap5 cells: min 5,8±1,1 µm; max 11,3±2,1 µm; P<0,005, and Ng2+cells: min 5,8±1,0 µm; max 10,7±1,5 µm; P<0,005).

Double staining with NeuN, β-tub, GFAP and Iba1, consistently excluded their neuronal, astroglial or microglial nature (Fig. 2B). This fact, along with morphological features described above, confirmed that mMap5 cells strongly resemble to a population of glial-like, synantocyte/polydendrocyte-like cells. They were widely distributed in all regions examined, interspersed among the Ng2+cells (Fig. 2C; detectable in mice but not in rabbits with immunocytochemistry, see Table 2 and [10]. As to their amount, mMap5 cells were far less numerous than the Ng2+cells, with an average proportion of 1∶30 (semi-quantitative evaluation on 407 Ng2+cells counted in 9 confocal images, from 3 animals, each containing at least one mMap5 cell; Fig. 2C). Double staining with Map5 and Ng2 in mouse did not reveal any overlapping (Fig. 2D), thus indicating that mMap5 cells are not a subpopulation of Ng2 progenitors (although this fact does not exclude they belong to the same lineage at a different differentiative stage).

**Table 2:** Primary antibodies used in this study.

Antigen	Antibody/antiserum	Host	Dilution	Source	Species tested
Map5	mono, clone AA6	mouse	1∶5000	Millipore	R,C,GP,S,Mn,H +, M −
	poly	goat	1∶300	Santa Cruz	M,R +
**Cell proliferation**					
Ki67	mono	mouse	1∶500	BD Pharmigen	M,R +
	poly	rabbit	1∶1500	Novocastra	M +, R −
BrdU	mono, BU1/75	rat	1∶300	AbDSerotech	M,R,C +
**Progenitors cells**					
SOX2	poly	rabbit	1∶1500	Millipore	M,R +
	poly	goat	1∶400	Santa Cruz	M,R +
SOX9	poly	rabbit	1∶1000	Millipore	M,R +
**Neuroblasts/Neurons**					
DCX	poly	guinea pig	1∶1000	Abcam	M,R +
	poly	rabbit	1∶1000	Abcam	M,R +
NeuN	mono, A60	mouse	1∶200	Millipore	M,R +
βIII-Tub	Mono (Tuj1)	mouse	1∶100	Millipore	M,R +
βIII-Tub	Poly (Tuj1)	rabbit	1∶1000	Covance	M,R +
**Astrocytes**					
GFAP	mono	mouse	1∶300	Millipore	M,R +
	poly	rabbit	1∶2000	Dako	M,R +
**Microglia**					
Iba1	poly	rabbit	1∶1000	Wako	M,R +
**Ependyma**					
S-100β	poly	rabbit	1∶10000	Swant	M,R +
**Oligodendroglia**					
***Precursor/immature***					
Ng2	mono	mouse	1∶200	Chemicon	M,R −
	mono	mouse	1∶500	US Biological	M,R −
	poly	rabbit	1∶200	Chemicon	M +, R−
	mono	mouse	1∶200	Sigma	M,R −
	mono	mouse	1∶200	Upstate	M,R −
OLIG2	poly	rabbit	1∶500	Millipore	M,R,C +, GP,S,Mn,H −
	poly	goat	1∶400	R&D System	M,R,C +, GP,S,Mn,H −
SOX10	poly	goat	1∶1000	Santa Cruz	M,R,C,GP,S,Mn +, H −
GPR17	poly	rabbit	1∶10000	Marta Fumagalli	M,R +
PDGFRα	poly	rabbit	1∶1000	Santa Cruz	M +, R −
		rat	1∶100	BD Pharmigen	M +, R −
JAM-A	poly	rabbit	1∶200	Santa Cruz	M −, R −
***Mature/myelinating***					
GST-π	poly	rabbit	1∶400	MBL	M +, R −
RIP (CNPase)	mono	mouse	1∶400	Chemicon	M,R +

**Figure 3:**
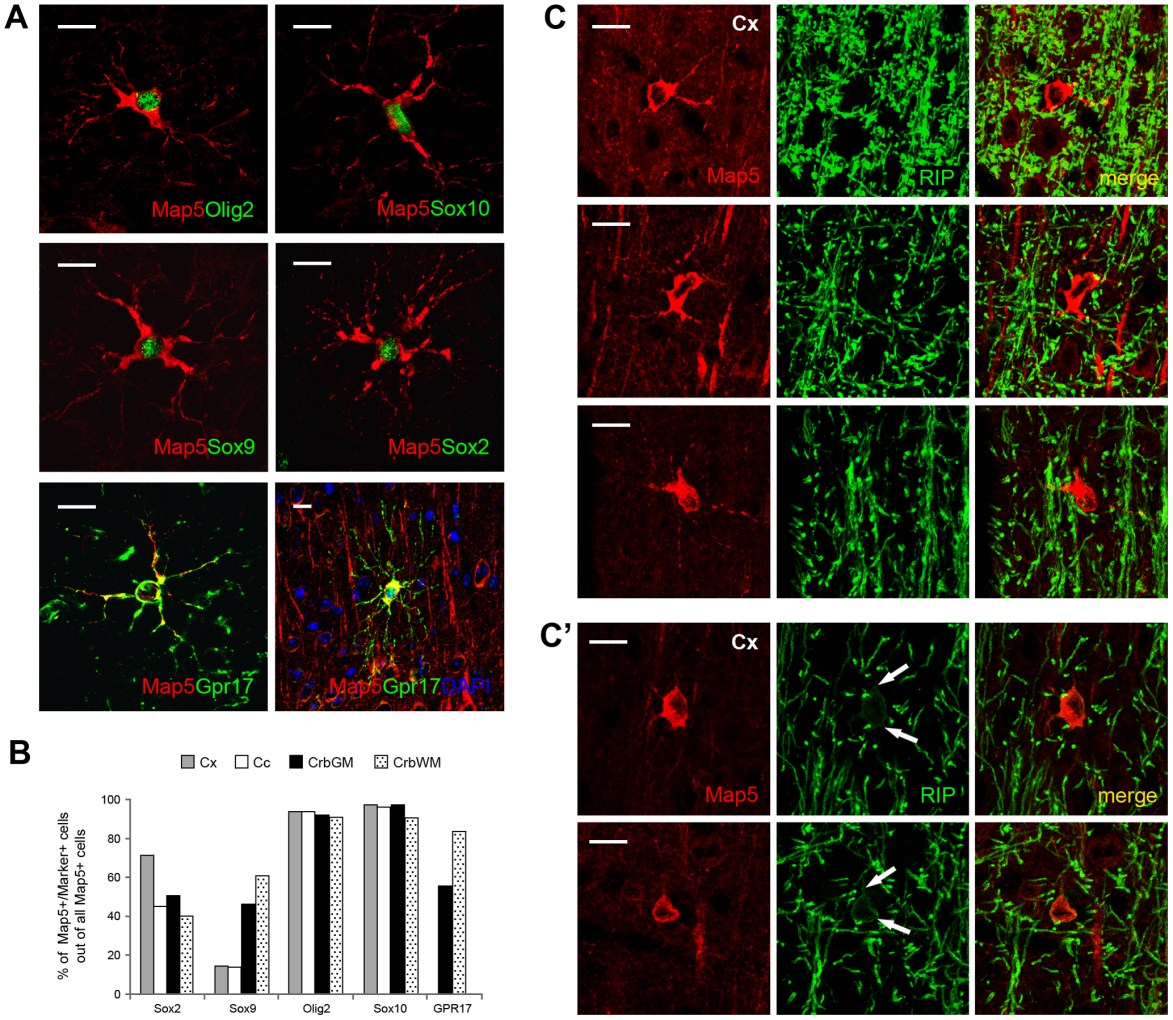
Phenotypic characterization of mMap5 cells in the oligodendrocytic lineage. A, Double staining between Map5 and different markers of the oligodendroglial lineage in mMap5 cells. B, Quantitative evaluation of mMap5 cells expressing different markers in different brain regions. C,C’, Very low overlapping between mMap5 cells and the mature oligodendrocytic population. Double staining with Map5 and mAb anti-RIP (CNPase) in the mouse cerebral cortex (Cx) generally showed no co-expression (C); a faint overlapping of the two antigens could be detectable only in some cells (C’, arrows). Scale bars: 10 µm.

To further study the phenotype of the mMap5 cell population, double and triple staining with different antigens of progenitor cells and oligodendroglial lineage differentiation stages were performed (Fig. 3). In all CNS regions examined the mMap5 cells were double stained with nuclear transcription factor Olig2 (Fig. 3), thus corresponding to elements of the oligodendroglial lineage. By using Olig2/Map5 double staining as a basis for recognizing the mMap5 cell population, the percentage of co-expression with other antigens were calculated (histograms in Fig. 3B). In addition to Olig2, the vast majority of the mMap5 cells do co-express the nuclear transcription factor Sox10, and a substantial proportion also express Sox2 and Sox9 (Fig. 3B; raw data in Fig. S3). The pattern of marker expression strongly indicates that mMap5 cells might represent a specific stage of maturation along the oligodendroglial lineage, even in the absence of overlapping between the Map5 and Ng2 markers. This is confirmed by the fact that a proportion of mMap5 cells (4–5% in cerebral cortex, 8 Map5+/GPR17+cells out of 179 Map5+cells, and corpus callosum of the adult mouse, 5 Map5+/GPR17+cells out of 122 Map5+cells; 56–84% in cerebellar grey matter, 55 Map5+/GPR17+cells out of 98 Map5+cells, and white matter, 42 Map5+/GPR17+cells out of 50 Map5+cells of the peripuberal rabbit) is immunoreactive for the new Ng2 cell marker GPR17 receptor [52] (Fig. 3B and S3), which has been shown to be present in 25–30% of Ng2+ cells in mice [53]. In most double staining GPR17 appear to label the membranes of the whole process ramifications, as previously described in more mature, premyelinating stages [53].

Finally, we performed a careful analysis on double staining with Map5 and RIP (a monoclonal antibody which recognizes the CNPase specific of mature oligodendrocytes; [54]. In most specimens, there was no co-localization (Fig. 3C), although a faint expression of Map5 was detectable in some oligodendroglial cells (Fig. 3C’). This suggests that Map5 expression precedes oligodendrocytes terminal differentiation.

### Experiments with cell proliferation antigens and BrdU pulse labeling followed by different survival times

**Figure 4:**
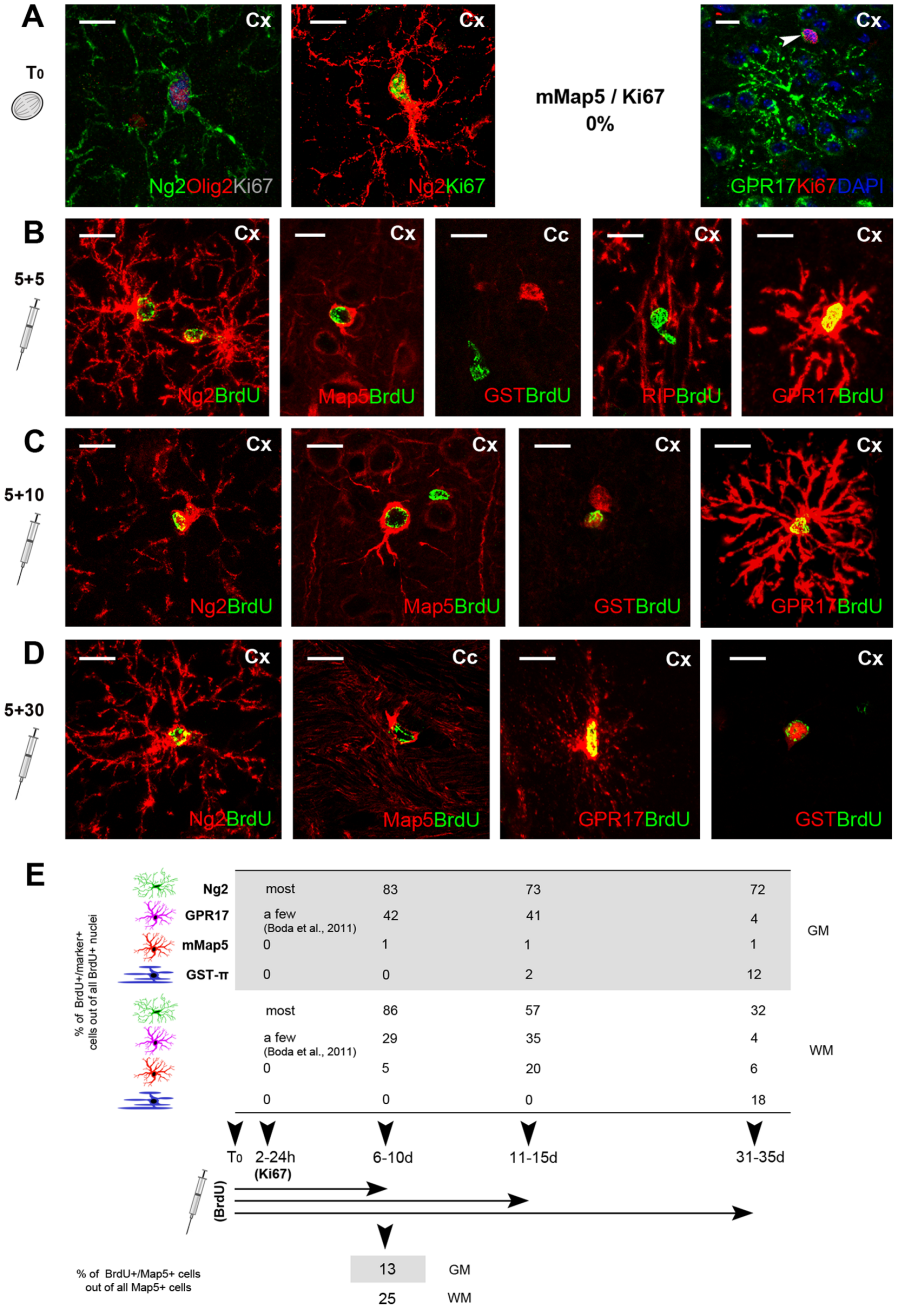
Genesis and differentiation of mMap5 cells in mice. Analyses carried out with endogenous and exogenous cell proliferation markers in double staining with Map5 and other markers of the oligodendroglial lineage. A, No proliferation of mMap5 cells is detectable by using the endogenous cell proliferation marker Ki67, whereas most Ng2+cells are clearly cycling. Rare Ki67+/GPR17+cells are detectable (A, right), their staining being restricted to the Golgi in the cell body (arrowhead). B–D, Analyses carried out with BrdU i.p. administration followed by different survival times (from 10 to 30 days). BrdU/Map5 double staining is detectable starting from 5–10 days in mice. E (top), quantification of newly generated cells expressing Map5, Ng2, GPR17, GST-π, at different BrdU post-injection survival times, in grey (GM, cortex) and white matter (WM, corpus callosum). Data indicate the percentage of BrdU+/marker+ cells out of total BrdU+ nuclei (raw data are reported in Fig. S3). E (bottom), quantification of newly generated mMap5 cells out of the mMap5 cell whole population (analysed in animals with 5+5 days BrdU treatment). Scale bars: 10 µm.

**Figure 5:**
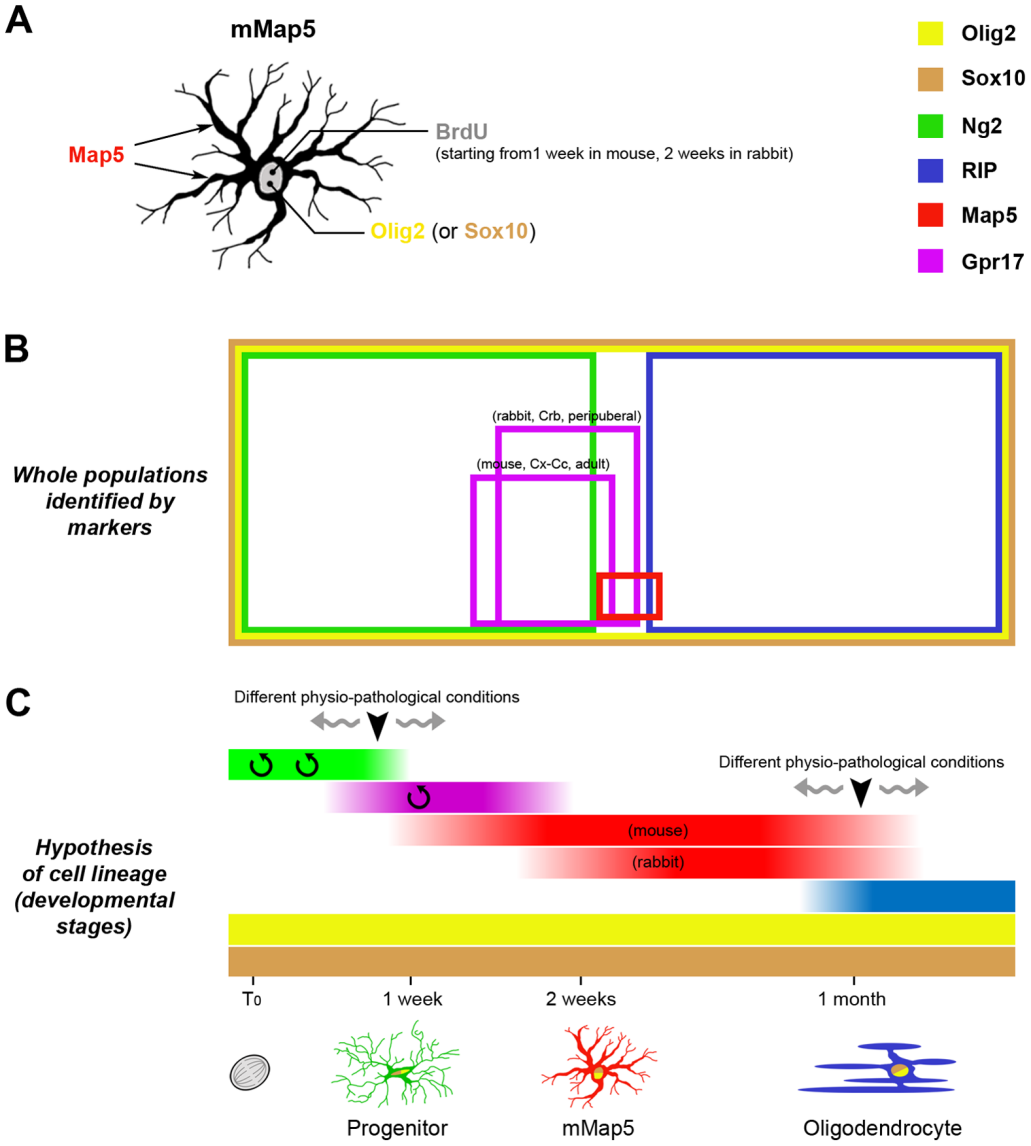
Schematic summary of the spatial and temporal distribution of several markers linked to the mMap5 cell population. A, The mMap5 cell population can be revealed by Map5/Olig2 double staining, and the subset of newly generated mMap5 cells by Map5/Olig2/BrdU triple staining (left). Double and triple staining with different markers (see colors in the legend on the right) can provide information on the spatial (B) and temporal (C) distribution of different subpopulations. B, The relative amount of each cell population revealed by different markers is indicated by colored squares. There is no overlapping between mMap5 and Ng2+cell population, and only a very small overlapping with the GST-π+ oligodendrocytes. C, Hypothesis on the time course for newly generated mMap5 cells by using endogenous and exogenous cell proliferation markers.

Since mMap5 cells display most antigens expressed at different stages of the oligodendrocyte precursor cell lineage [55], [56], we performed a set of experiments with cell proliferation markers in order to gain more insight on their putative origin from cycling progenitors. Double staining carried out in different CNS areas with Map5 and Ki67 antigen (a cyclin expressed in cells during the G1, S, G2 and M phases, but not in G0, thus in all proliferating cells, with a peak during DNA synthesis and mitosis [57]) were consistently negative (Fig. 4A). By contrast, and as expected, double staining between Ki67 antigen and Ng2 frequently revealed cycling Ng2+progenitors (Fig. 4A). Ki67+/GPR17+cells were rare and, when occurring, they showed a typical staining of the Golgi (restricted to the cell body and absent in processes [53]; Fig. 4A). By analysing BrdU-injected mice killed at different survival times (from 1 week to 1 month, see methods; Fig. 4B–D), the first BrdU+/Map5+double labelling was observed starting from 6–10 days (Fig. 4B). In parallel experiments, carried out on rabbits, the first BrdU+/Map5+double labeling was detectable at 11–15 days survival (not shown). Hence, mMap5 cells are newly generated in the adult mammalian CNS, yet postmitotic when expressing this microtubulin protein, which appears 1 or 2 weeks after cell division, depending on the animal species. Counting of the number of BrdU+ mMap5 cells out of all BrdU+ nuclei (percentage of newly generated mMap5 cells out of all newly generated cells) at different survival times, in the cerebral cortex and corpus callosum (Fig. 4E, top and Fig. S3) revealed that they are stable at low levels in the grey matter, whereas they increase in the white matter at 1–2 weeks, then decreasing again at 1 month. Then, we counted the percentage of BrdU+ mMap5 cells out of all mMap5 cells, at the time they start to be detectable as newly generated cells in mice (end of the first week; see Fig. 4E, bottom). We found that 13% (7 Map5+/BrdU+ cells out of 53 Map5+cells, counted from 3 mice) and 25% (16 Map5+/BrdU+ cells out of 63 Map5+cells, from 3 mice) of the mMap5 cells are newlyborn, respectively in the grey and white matter. Finally, we quantified the percentage of newly generated cells immunoreactive for Ng2, GPR17, or GST-π (number of BrdU+/marker+ cells out of all BrdU+ nuclei) in order to compare them with those of the BrdU+ mMap5 cells (data summarized in Fig. 4E). As expected, most of the Ng2+cells and a substantial percentage of the GPR17+cells display active proliferation and appear to be newly generated during the first two weeks. At the same time, GPR17 expression is high (thus providing a link between Ng2+and mMap5 cells), then droppig fastly. At 1 month survival, beside a high persistence of newlyborn Ng2+cells (more evident in the grey than white matter, wherein they drop from 86% to 57% to 32%; see Fig. 4E) only a few newlyborn GPR17+cells can be found (4% in both grey and white matter). Conversely, only a few cells with features of mature oligodendrocyte are detectable at short periods after BrdU injection, whereas they dramatically increase at 1 month. Taken together, results from the time course experiments show that mMap5 cells are largely newly generated, postmitotic elements of the oligodendroglial lineage. Furthermore, they indicate that some mMap5 cells can differentiate into mature oligodendrocytes (especially and more rapidly in the white matter) or maintain the Map5 staining for undetermined times (at least 1 month; see Figs. 4E and 5C).

### Regional distribution of mMap5 cells in the CNS of rabbits and mice

**Figure 6:**
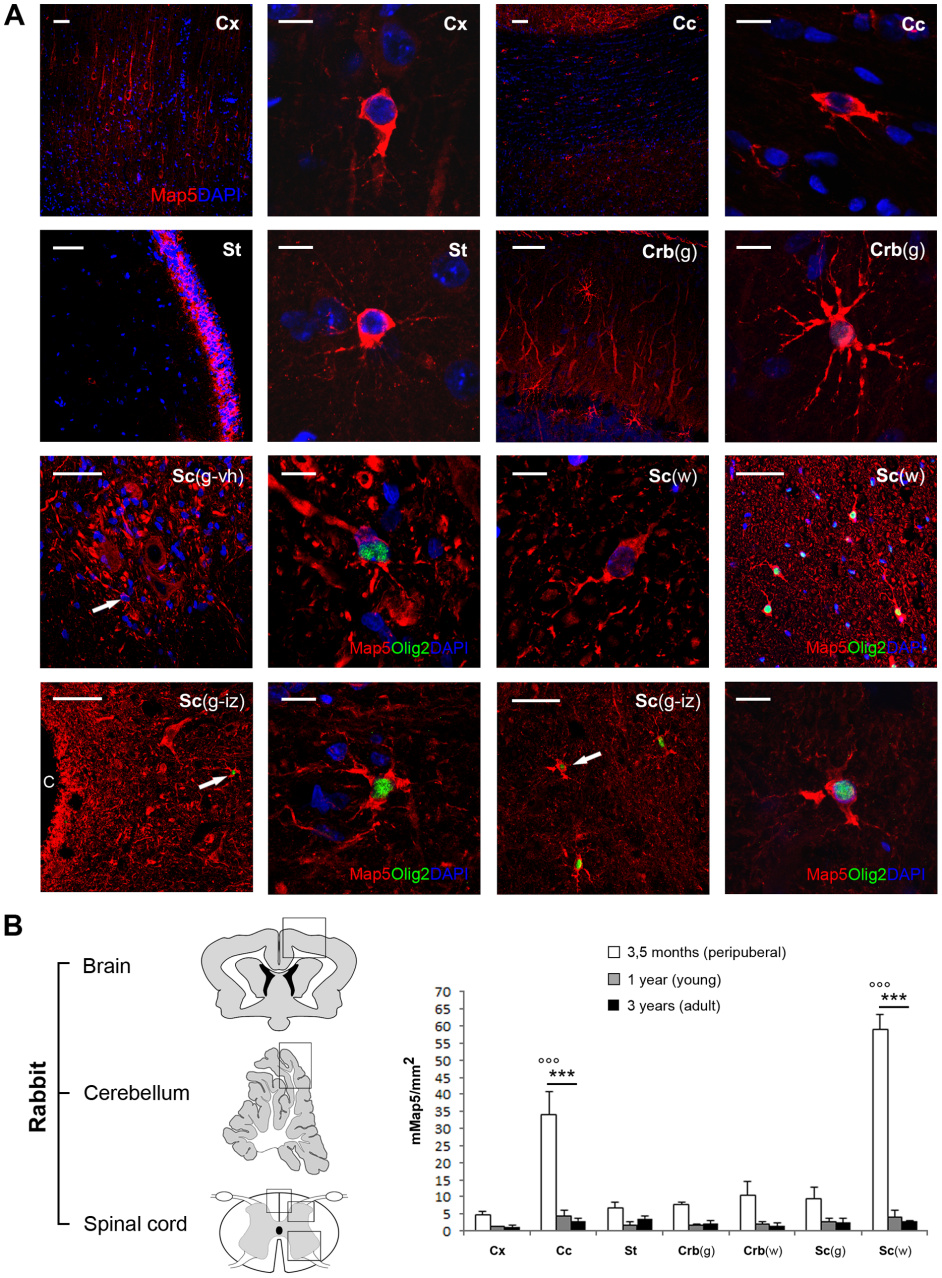
Regional distribution of mMap5 cells in the rabbit CNS. A, Representative images of mMap5 cells in the cerebral cortex (Cx), corpus callosum (Cc), striatum (St), cerebellum (Crb), and spinal cord (Sc). g, grey matter; w, white matter; vh, ventral horn; iz, intermediate zone; c, central canal. B, Quantitative analysis of mMap5 cell density in different regions, at three different ages. Squares indicate the areas in which cell counts have been carried out. Asterisks/dots indicate significant statistical differences in mMap5 cell densities among different regions/ages (two way ANOVA, p<0,001). Scale bars: Low magnifications, 50 µm; high magnifications, 10 µm.

**Figure 7:**
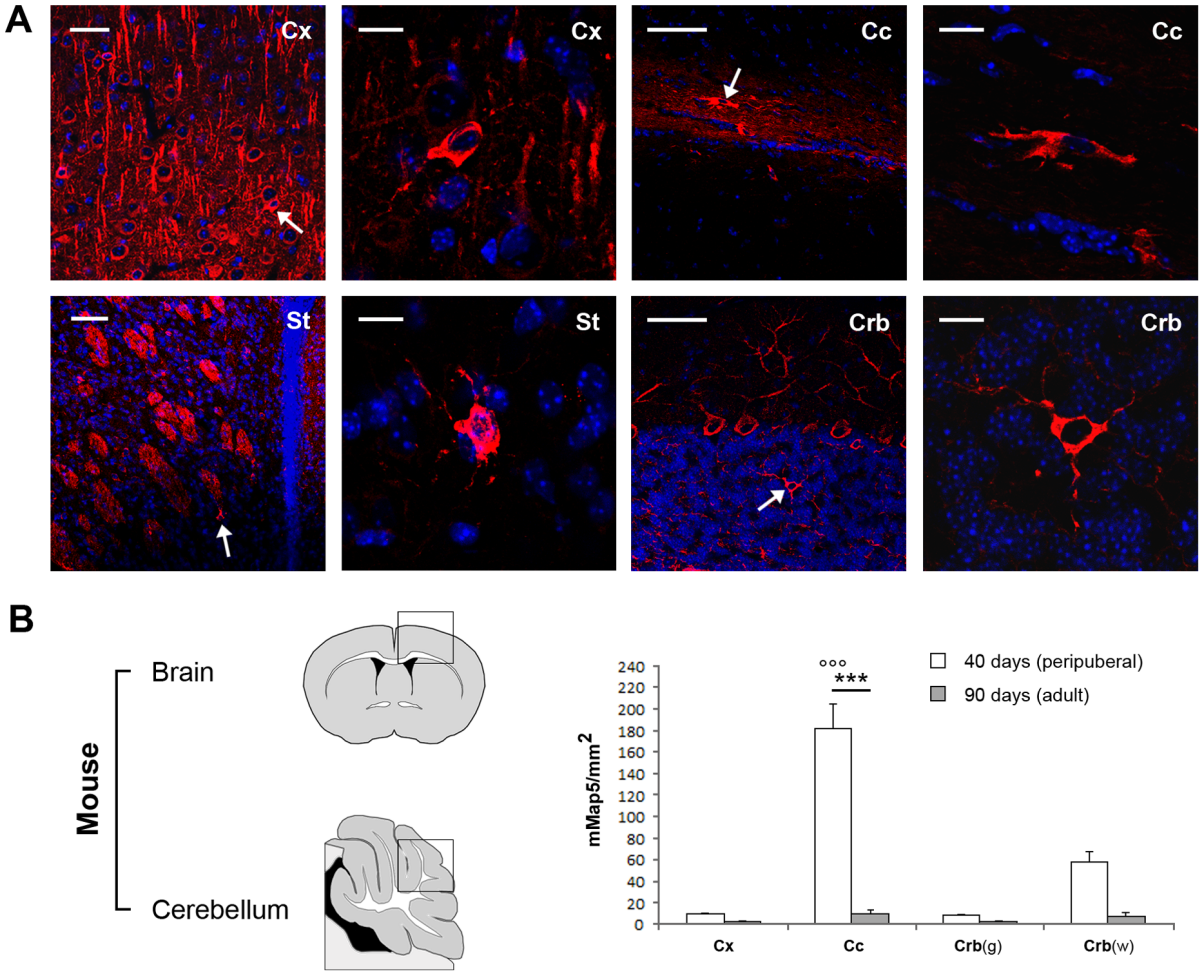
Regional distribution of mMap5 in the mouse CNS. A, Representative images of mMap5 cells in the cerebral cortex (Cx), corpus callosum (Cc), striatum (St), cerebellum (Crb). g, grey matter; w, white matter. B, Quantitative analysis of mMap5 cell density in different regions, at two different ages. Squares indicate the areas in which cell counts have been carried out. Asterisks/dots indicate significant statistical differences in mMap5 cell densities among different regions/ages (two way ANOVA, p<0,001). Scale bars: Low magnifications, 50 µm; high magnifications, 10 µm.

In order to verify if mMap5 cell occurrence and distribution is widespread in the CNS and if it parallels the heterogeneity reported in literature for the synantocyte/polydendrocyte populations, qualitative and quantitative analyses were carried out in several CNS regions of the rabbit (cerebral cortex, corpus callosum, striatum, cerebellum, spinal cord; Fig. 6) and mouse (cerebral cortex, corpus callosum, cerebellum; Fig. 7). These regions were chosen since they include both white matter tracts and grey matter parenchyma. As stated above (see also Fig. 1B), mMap5 cells are far less detectable in mouse tissue with respect to rabbit, due to a different staining generally more restricted to the cell body and primary processes in rodents (Fig. S2A). For this reason, in parallel with regional analysis in mice (Fig. 7), we performed a more widespread study in different areas of the rabbit CNS (Fig. 6). Morphological variations in the overall cell shape were observed, with particular differences in white and grey matter. Most mMap5 cells had a round soma from which 3–4 main processes radiate in all directions. In grey matter they were arranged among the neuronal cell bodies, and their processes occupied a roughly spherical territory (Figs. 6 and 7). In the cerebral cortex (both six- and three-layered regions) the mMap5 cells were often resting on neuronal somata and processes (Fig. 1A, bottom). Quantification of mouse cell soma diameters in this region is reported in Fig. S2B. In the cerebellum they were associated with Purkinje cell somata, in this case assuming a more flattened shape, or widespread in the cortex with a soma prevalently round-shaped (quantification of mouse cell soma diameters in Fig. S2B). In white matter (e.g., corpus callosum and spinal cord fiber tracts), the cell bodies were more flattened/elongated with few short processes starting from the two poles and trapped in-between myelinated axons (Figs. 1B and 6A; quantification of mouse cell soma diameters in Fig. S2B), which are reminiscent of the GPR17+cells detected by Lecca et al. [58]. The most ramified, typically stellate morphology was frequently detectable in the granule layer and molecular layer of the cerebellum, and to a lesser extent in the striatum. mMap5 cells in the cerebral cortex and spinal cord grey matter were more irregular/heterogeneous in their overall shape, frequently endowed with three main processes (Fig. 6A, bottom). In the spinal cord grey matter mMap5 cells were more abundant in the intermediate zone, less abundant in the dorsal horn, and rare in the ventral horn (Fig. 6A, bottom). The wider variety of different shapes was detectable in the spinal cord white matter, with many bipolar and neuronal-like, pyramidal morphologies (Fig. 6A, bottom). These shapes were unusual with respect to the multipolar aspect of mMap5 cells in other regions.

Hence, some differences (more flattened in white matter, more multipolar in grey matter) substantially reflect those described for the Ng2+cells [1]. Nevertheless, our analysis of cell soma diameters revealed higher heterogeneity for mMap5 cells in some CNS areas with respect to that described for Ng2+cells (see Fig. S2B).

In order to assess the number of mMap5 cells in different CNS regions, cell densities were counted in all the areas examined, at different ages. Quantification results are reported in histograms of Fig. 6 (rabbit) and 7 (mouse). In general, mMap5 cells are quite numerous in the white matter with respect to grey matter, both in rabbits and mice (see statistics in Figs. 6 and 7). Yet, this difference is remarkable only in young animals, since the number of mMap5 cells in the white matter falls after puberty (see statistics in Figs. 6 and 7, and below for age-related considerations). This substantial reduction of mMap5 cell density in the shift from the peripuberal to adult period is particularly evident in large bundles of axons of the corpus callosum and spinal cord dorsal columns. The age-related drop is more evident in mice, due to a higher initial cell density in rodents with respect to lagomorphs. At subsequent ages the cell densities seem quite stabilized, even in old animals (e.g., in 1-year old mice; value referred to a different strain).

### Comparative analysis of mMap5 cells occurrence in other mammals

**Figure 8:**
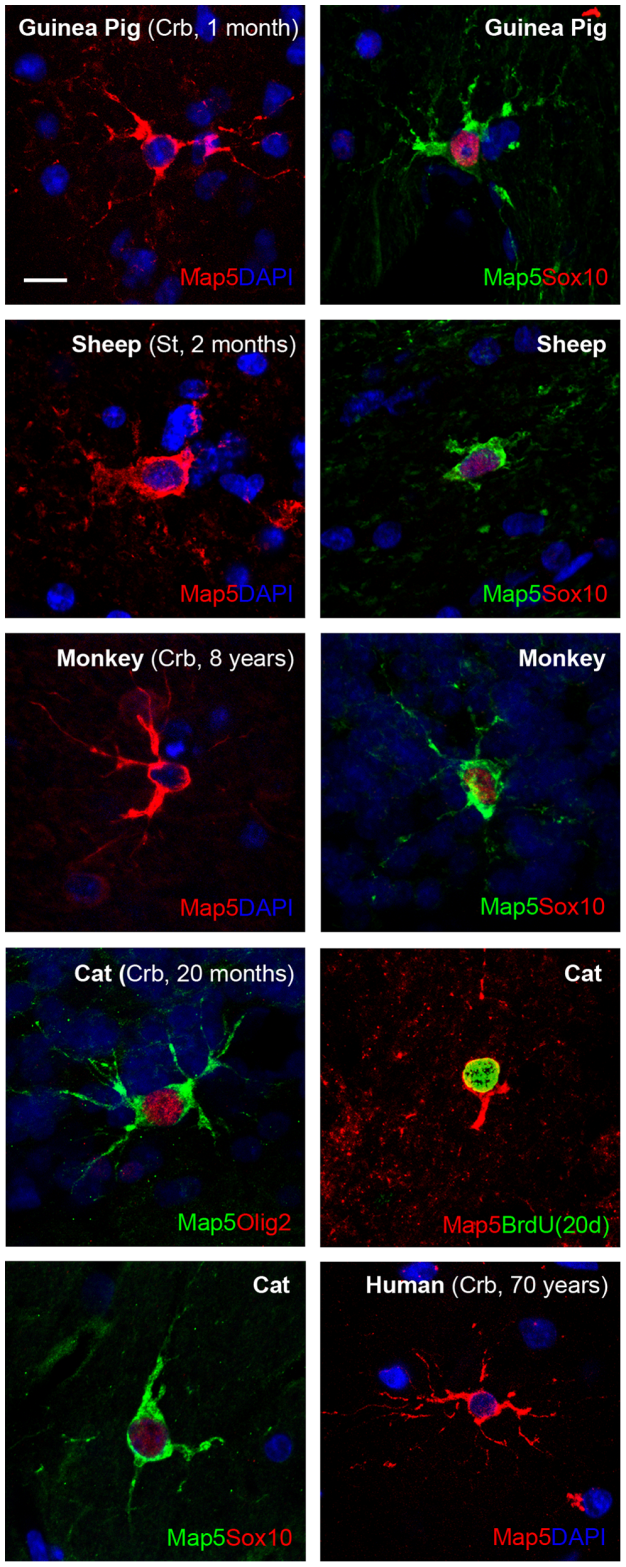
Occurrence of mMap5 in different mammals. Multipolar, Map5+cells showing the same morphology and set of markers found in mouse and rabbit are detectable in various CNS regions of different mammals, including humans. Scale bars: 10 µm.

In addition to the systematic analysis in most regions of the rabbit CNS and mouse brain, we analyzed brain tissue sections from other mammalian species characterized by increasing brain size and neocortex complexity (guinea pig, cat, sheep, monkey, human; Fig. 8). Different regions, including cerebral cortex, striatum, cerebellum, and different ages, up to 70 years in humans, were considered. Multipolar Map5+cells were found in all species, regions and ages investigated. In most cases, compatibly with tissue fixation/antibodies successful staining, these cells showed co-expression with markers tested in rabbit and mouse, thus confirming they belong to the mMap5 cell population (Fig. 8). The wide range of presence of mMap5 cells in the CNS of different mammals indicates a common role/distribution in phylogeny, including humans.

### mMap5 cell distribution in chronic neurodegenerative damage and in acute trauma

**Figure 9:**
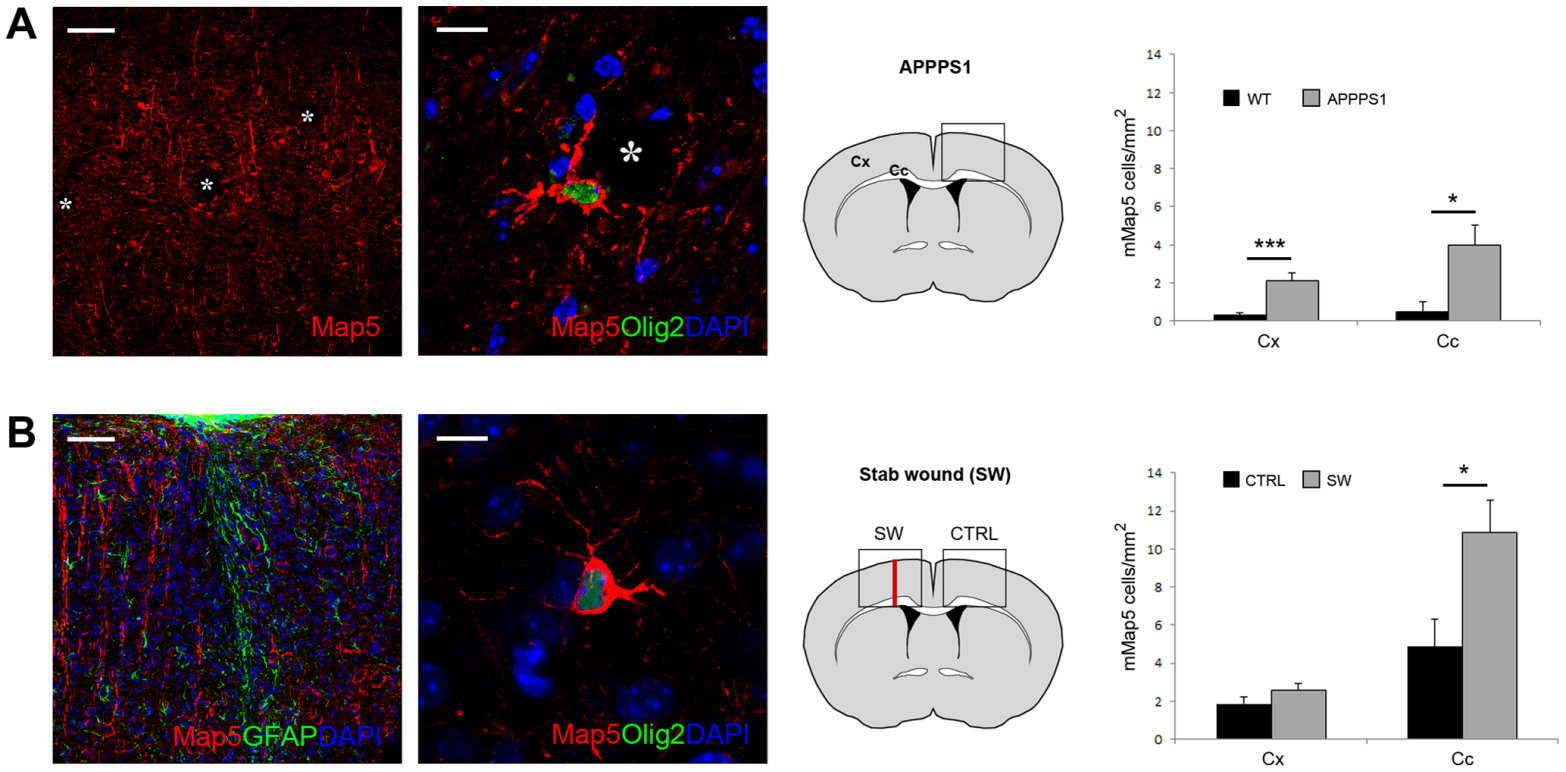
Behavior of mMap5 cells under neurodegenerative and traumatic injury conditions in mice. A, The number of mMap5 is significantly increased in the cerebral cortex (Cx) and corpus callosum (Cc) of APPPS1 trangenic mice. Asterisks, amyloid plaques. B, A slight increase in the amount of mMap5 is detectable after stab wound lesion in the mouse cerebral cortex and corpus callosum. Differences between A and B in the number of mMap5 in WT animals is related to the different ages at which the two lesion models were analysed (12 months for Alzheimer and 3 months for stab wound). Scale bars: Low magnifications, 50 µm; high magnifications, 10 µm.

Although many reports indicate the general involvement of Map5 molecule in different pathological conditions [40], the participation of mMap5 cells is still unknown. To gain more insight into the role of mMap5 cells in chronic damage we extended our analysis to a mouse transgenic model of neurodegeneration (APPPS1, a model of Alzheimer's Disease; [59]. Notably, the density of Olig2+mMap5 cells was significantly increased in the APPPS1 cortical grey matter compared with age-matched WT controls (2,1±0,4 cells/mm^2^ in APPPS1 versus 0,3±0,1 cells/mm^2^ in WT, p<0.001; Fig. 9A). The number of Olig2+mMap5 cells was also significantly increased in the white matter of the corpus callosum (4±1 cells/mm^2^ in APPPS1 versus 0,5±0,5 cells/mm^2^ in WT, p<0.05; Fig. 9A). To address the issue of a possible involvement of mMap5 cells in acute trauma conditions, we analyzed the cerebral cortex and corpus callosum of mice after stab wound (SW) injury. At 15 days post-lesion the density of Olig2+mMap5 cells in the grey matter was similar to that detectable in intact mice (2,6±0,2 cells/mm^2^ in SW, versus 1,9±0,4 cells/mm^2^ in control, p>0.05; Fig. 9B). By contrast, the number of Olig2+mMap5 cells was significantly increased in the underlying white matter (10,8±1,8 cells/mm^2^ in SW versus 4,9±1,7 cells/mm^2^ in control, p<0.05; Fig. 9B). This result is consistent with previous studies reporting that GPR17, which is also expressed by these Olig2+mMap5 cells, is significantly increased in both the APPPS1 model and after induction of SW in rodents [53].

Hence, our data show that a substantial increase of mMap5 cells does occur in chronic neurodegenerative conditions (both in grey and white matter), and, to a lesser extent, after traumatic injury (in the white matter only).

### Map5 expression and mMap5 cells at different ages

**Figure 10:**
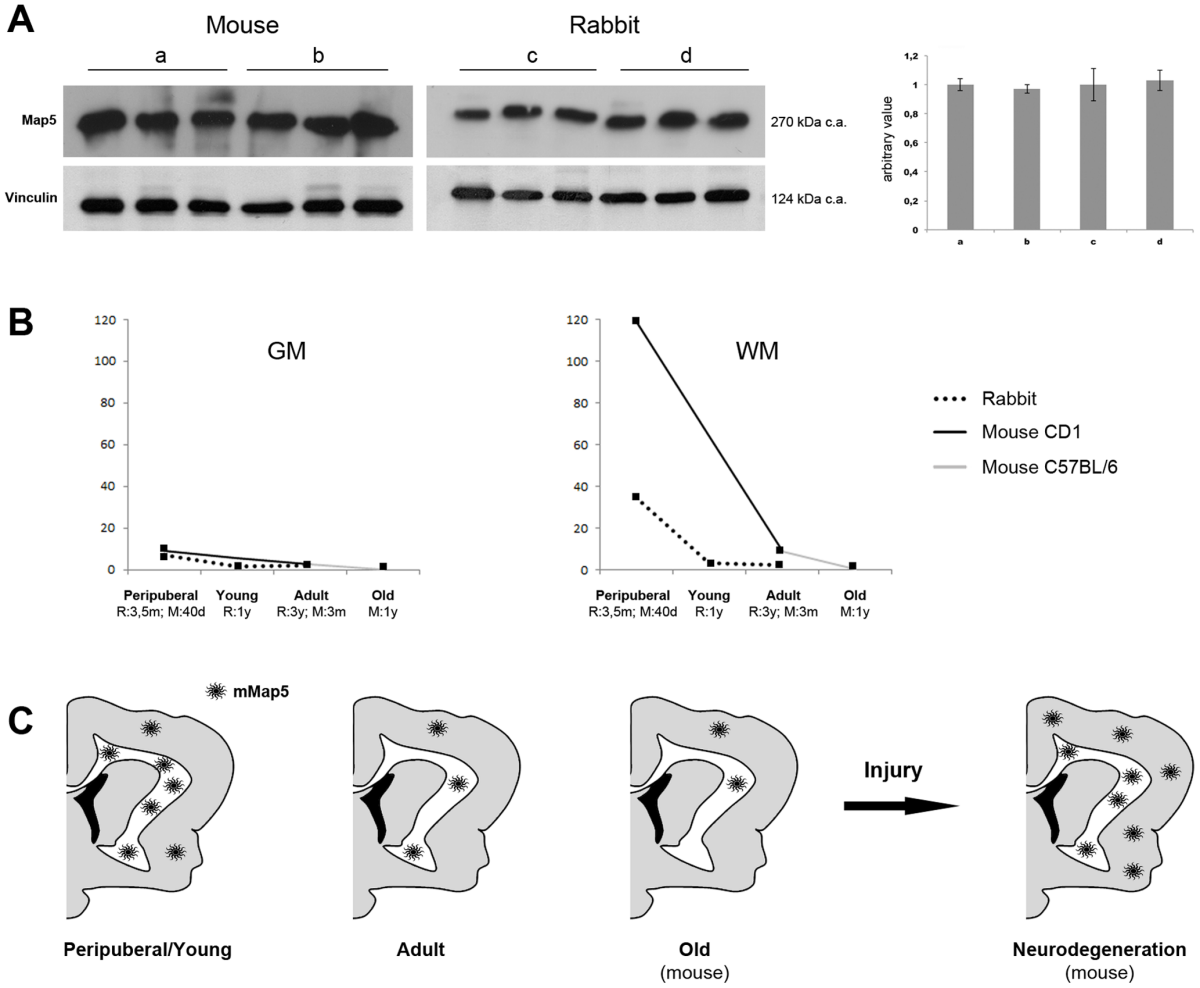
Amount of Map5 molecule and mMap5 cells in the rabbit and mouse CNS at different ages. A, Immunoblot analysis of Map5 (Map1b) expression in different animals at different ages. Autoradiography of the western blot of protein lysate from total brain of mouse 40 days old (a) and 3 months old (b), rabbit 3,5 months old (c) and 1 year old (d), probed with anti-Map5 and anti-vinculin antibodies (3 animals for each condition). On the right, quantitation of the image after normalization with vinculin. The values expressed are a media of the value of the 3 animals for each condition. Values of older animals were expressed relatively to those of young animals of the same species. Level of Map5 in the brain of the older animals does not change significantly with respect to level in the young animals of the same species. B, Trend in the amount of mMap5 cells in the rabbit and mouse CNS at different ages. C, Schematic representation of the data reported in B, and reactivity of mMap5 after injury.

Since the amount of polydendrocytes remarkably varies with age [60], [61], we analysed the behavior of mMap5 cells at different postnatal (peripuberal and adult) stages (Fig. 10). First of all, western blots of protein lysate from total brain of mouse (40 days and 3 months old), and rabbit (3,5 months and 1 year old), probed with anti-Map5 and normalized with anti-vinculin antibodies, were performed. Values (from older animals expressed relatively to young animals of the same species) indicated that total level of Map5 did not change significantly with respect to age (Fig. 10A). This result must be read as the total amount of neuronal and glial Map5, although we know that it is prevalently expressed in neurons (see first paragraph of the Result section and Fig. 1). By contrast, counting of the mMap5 cells at different ages clearly indicated their progressive reduction from peripuberal to adult stages, more dramatic in the white matter (regional data in rabbit and mouse are reported in Figs. 6 and 7; the trend of average data from all regions examined is summarized in Fig. 10B). Their level then results stabilized during adulthood and in old individuals (the latter data are referred to 1 year old mice of the C57BL/6 strain). On the whole, by comparing Map5 expression in peripuberal and young/adult rabbits with age matched mice, in both species we observed no significant reduction of the total Map5 molecule amount with age (Fig. 10A), but significant reduction of the mMap5 cell number, especially in the white matter (see graphics in Fig. 10B, and rabbit, mouse regional data in Figs. 6 and 7).

## Discussion

In the adult mammalian CNS, beside *de novo* cell genesis of ‘classic’ neurogenic sites (SVZ and dentate gyrus [62], [63]), the occurrence of local, parenchymal progenitors which continue to divide throughout life has progressively became a new area of investigation [1], [2], [64], [65]. In vivo, these cells are different from neural stem cells, since they are not segregated within germinal layer-derived, highly regulated niches [66], [67]. Parenchymal progenitors are widespread in the CNS tissue, so that they do not need to migrate to reach the injured sites (see [3] for review). They can react to brain injury in different ways [68], [69], [70], whereas neurogenesis supported by the stem cell niches appears functionally committed to restricted physiological roles and poorly supportive for repair [71], [72].

In spite of the indisputable interest for parenchymal progenitors, our knowledge of their biology remains incomplete due to a remarkable difficulty in defining cell populations/subpopulations which often display regional features and different degrees of differentiation, not always revealed by specific markers. Here we analysed the cellular and molecular features of a subset of multipolar cells immunoreactive for Map5, a microtubule-associated protein expressed in both neurons and glia [40]. In the past, the glial localization of Map5 has been underestimated. This fact could be attributable to the different appearance of mMap5 cells in mice and rabbits described here, which likely contributed to the ‘hiding’ of mMap5 cells in previous studies carried out on rodents (see for example [13], [29]).

### mMap5 cells are a population of newly generated, postmitotic parenchymal elements of the oligodendroglial lineage

Although widely expressed in different types of neuronal cells (mature neurons and neuroblasts generated from both neural stem cells and parenchymal progenitors), Map5 also decorates a subset of multipolar glial-like cells (Fig. 1). We show that these multipolar elements, here referred to as mMap5 cells, are not neurons, nor astrocytes or microglia, and consistently express Olig2, Sox10, and, to a lesser extent, Sox9 and Sox2 transcription factors in their nuclei (Figs. 2 and 3). Olig2 is necessary for oligodendrocyte lineage development in multipotent progenitor cells of the embryo and adult [73]. The transcription factors Sox9 and Sox10 jointly occur in cells of the oligodendrocyte lineage, being important for their specification and terminal differentiation [74]. Sox9, also expressed in astrocytes, in the oligodendrocyte lineage marks the stage of immature oligodendrocyte precursors, then being turned off during maturation. Sox10 is not expressed in all CNS glial cells, being restricted to the oligodendrocyte lineage. With the onset of specification, Sox10 starts to be expressed in proliferating oligodendrocyte progenitors and persists in mature, terminally differentiated oligodendrocytes (reviewed in [74]). On the whole, the pattern of molecular expression found in mMap5 cells clearly places them in the oligodendroglial lineage. In addition, some mMap5 cells also express Sox2, a transcription factor involved in neural stem/progenitor cell maintenance [75]. This raises the question if either all mMap5 cells are committed to the oligodendroglial lineage or some of them might retain properties of immature/progenitor cells. To answer this question, we performed a study on cell proliferation dynamics, by using different cell division markers (Fig. 4). Our results obtained with the endogenous cell proliferation marker Ki67 demonstrate that mMap5 cells are not cycling elements (as the Ng2+progenitors are [1], [2]). Nevertheless, BrdU injections followed by different survival times showed that, even if postmitotic, when expressing the Map5 molecule a substantial proportion of these cells is newly generated (25% of all the mMap5 cells and 20% of cells which are newly generated at 10–15 days post-injection; data detected with our BrdU injection protocols, which cannot reveal all the newlyborn cells and thus underestimate the total number of newly generated mMap5 cells). It is interesting to note that, unlike Map5 expression in neurons, which spans from progenitors of the ventricular zone to fully mature neurons (our data in the adult, but see also [21] in the embryonic ventricular zone), in the glial lineage Map5 is expressed in newly generated cells, but only when they are postmitotic. In other words, although mMap5 cells are not actively proliferating, new mMap5 cells are generated from cycling cells, thus inserting them in a stage-specific state of oligodendroglial-like precursors (deriving from parenchymal progenitors, as confirmed by co-expression of GPR17; see below).

Beside morphological and molecular aspects strongly reminiscent of sinantocytes/polydendrocytes [1], [2], [5], the mMap5 cells also show different features: their processes are quite smooth, without swelling and varicosities typical of Ng2+cell processes [1]; such difference is qualitative and linked to the cytoskeletal nature of Map5 with respect to the membrane-bound localization of Ng2. Due to the high number of Ng2+cells with complex and overlapping processes, an in vivo quantification of ramifications could be unreliable. In addition, the cytoskeletal protein Map5 could not fill the whole extent of cell process ramifications. For these reasons we chose to quantify the cell somata diameter of both mMap5 and Ng2+cells. Soma diameters of Ng2+cells were prevalently elongated and rather constant in all regions, whereas those belonging to mMap5 cells are more heterogeneous, being prevalently round-shaped in grey matter. As expected, the average soma diameter was not significantly different in the three regions and its mean value (8,9±0,1 µm) is very similar to that recently reported for a subset of premyelinating cells (9,7±0,9 µm; detected in Lucifer yellow-filled cells in PLP-GFP mice [50]). Taken together, these features suggest that mMap5 cells could be elements which derive from parenchymal progenitor cell division, yet being in a non-cycling, more stable and specialized/differentiated stage.

Even though Map5 and Ng2 never co-localize, mMap5 cells may represent a small population of more mature, ‘intermediate’ cells deriving from Ng2 precursors that have already dowregulated this proteoglycan during their differentiation pathway. Support to this view comes from the expression analysis of GPR17, a membrane G-protein coupled receptor recently deorphanized [76], which has been shown to represent a new Ng2 cell marker [52] and to occur in oligodendrocyte precursor cells [58] as a transiently expressed functional modulator [52], [53], [77]. In detail, GPR17 has been shown to decorate two distinct differentiation stages of Ng2+cells: an early stage of still proliferating cells expressing GPR17 mainly in the cell body along with other precocious markers (e.g., platelet-derived growth factor receptor alpha, PDGFRα), and a more advanced post-mitotic stage, at which cells are losing Ng2 but GPR17 is maximally upregulated in cell processes [52], [53]. Our findings show that GPR17 is also detectable in a proportion of mMap5 cells (Figs. 4 and 5B), thus suggesting that Map5 specifically labels this second transition state (see also below) and is most probably expressed in-between two distinct stages of differentiation (Fig. 5C). GPR17 is upregulated in cells which assume premyelinating features, after having lost Ng2 and having expressed the receptor in the cell processes [53]. Accordingly, we found GPR17 cellular localization in somata and processes of mMap5 cells, which is, again, consistent with a more mature stage.

In order to provide a more complete picture of all newly generated cells in the oligodendroglial lineage, we extended the time-course experiments to the relative markers. In mice injected with BrdU and killed at different survival times, we analyzed and quantified the percentages of co-expression between the proliferation marker and different oligodendroglial cell markers (Fig. 4). Map5 does not colocalize with BrdU prior than 10 days post injection, thus confirming that at least 1 week is necessary before its expression in newly born mMap5 cells (2 weeks in rabbit, as found in parallel experiments carried out on this species). Starting from the second week, the percentage of Ng2+/BrdU+ double labeled cells decreases in parallel to an increase in the number of BrdU+ mMap5 cells. This trend confirms that new mMap5 cells, although not actively proliferating, are generated from cycling cells (most likely Ng2+progenitors). On the other hand, the increase in percentage of cells co-expressing BrdU and the mature oligodendrocyte marker GST-π detectable at subsequent times, suggests that some mMap5 cells might differentiate into oligodendrocytes. It is important to note that both decrease of Ng2+newly generated cells and increase of mMap5 newly generated cells are far more visible in the white matter (while almost stable in the grey matter; see Fig. 4E). This pattern supports the hypothesis that mMap5 cells are an intermediate stage between Ng2+progenitors and oligodendrocytes in the white matter, yet leaving open possibilities for other roles in the grey matter.

### mMap5 cells are reduced in number during adult/old ages but can increase after brain damage

Previous studies indicated that Map5 expression is high during development and at peri-natal stages [11], [40], then undergoing an early post-natal reduction to persist at lower levels in the peripuberal and adult CNS (see for example quantitative data on western blots from rat cerebral cortex and spinal cord, in [37]). Here, we confirm that the total amount of Map5 molecule in brain lysates remains substantially unchanged during adulthood, a trend which is clearly due to the prevalent expression of the protein in mature neurons throughout the CNS, as also observed qualitatively in immunocytochemical specimens.

In parallel to the total Map5 expression, as shown by our cell counts, the mMap5 glial cells do represent a very small cell population, even smaller that the NG2+cells (about 1∶30; see Figs. 2 and 5). Quantification of the mMap5 cell densities in both mouse and rabbit, revealed that their number decreases dramatically during the peripuberal period (Figs. 6, 7 and 10), at later stages with respect to that observed for neurons, being likely related to the last phases of myelination [78]. Indeed, the drop in mMap5 cells is particularly evident within large white matter tracts, and by comparing the two mammalian species, it starts from absolute values which are higher in mice, then following a similar trend although more diluted in rabbits (Fig. 10B).

Our data indicate that even if the number of mMap5 cells becomes stabilized at low levels at adult/old ages, in some injury/pathology situations their number can increase again (Fig. 10C). This increase seems to be more evident in chronic neurodegenerative states with respect to traumatic events, and, surprisingly, in grey matter with respect to white matter (Fig. 9). In this context, it is interesting to note that while the dramatic decrease observed during postnatal development does occur mainly in white matter (likely linked to the last phases of myelination), the increase detected in APPPS1 mice is higher in the cerebral cortex grey matter. This fact, even taking into account that neurodegenerative lesions are less evident in the white matter, opens the possibility that mMap5 cells could subserve other functions within the lesioned parenchyma. However, the more prominent increase observed in chronic neurodegeneration with respect to acute trauma could be linked to temptative myelinization.

In conclusion, we show that mMap5 cells represent a subset of newly generated cells in the CNS parenchyma. These cells occur in the nervous tissue of most mammalian species, including humans, wherein they can be easily identified with a stage-specific marker, thus allowing further studies on their behavior in different physiological and pathological conditions.

## Methods

### Animals and tissue preparation

All experimental procedures were in accordance with the European Communities Council Directive of 24 November 1986 (86-609 EU) and the Italian law for the care and use of experimental animals (DL.vo 116/92). Experiments carried out in this study were approved by the Italian Ministry of Health (8 october 2009) and the Bioethical Committee of the University of Turin. Mice and rabbits were raised in the NICO animal facility (authorization n. 182/2010-A). All experiments were designed to minimize the numbers of animals used and their discomfort. Six peripuberal (3,5 months old), three young (1 year old) and three adult (3 years old) female New Zealand White (*Oryctolagus cuniculus*), 3 peripuberal (40 days old) and 12 adult (3 months old) CD-1 mice (*Mus musculus*), were used. In addition, to extend the comparative analysis, some sample tissues of other mammalian species were provided through collaborations with other Institutions. Fixed brain sections were obtained from: one female albino Dunkin-Hartley guinea pig (*Cavia porcellus*, 30 days old) provided by Dpt. of Animal and Human Biology, University of Turin (authorization of the Italian Ministry of Health number 66/99-A); one adult cat (*Felis silvestris catus*, 18 months old) provided by Dept. de Fisiología y Zoología, Sevilla, Spain (Servicio de Animales de Experimentación, Universidad de Córdoba, Spain; approved by the Committee on Bioethics of the València University (R.D. 120/2005 BOE 252/34367-91, 2005); one young female sheep (*Ovis aries*, 2 months old) provided by Istituto Zooprofilattico Sperimentale del Piemonte, Liguria e Valle d'Aosta (post-mortem material collected at a local slaughter house: Old Bear S.a.s. Cuneo, Ronchi, Italy); one adult male macaque monkey (*Macaca fascicularis*, 8 years old) provided by Dpt. of Physiology and Pharmacology of the University of Rome Sapienza, Italy (authorization of the Italian Ministry of Health 27 september 2010; the animal was housed not in single cage but in a large common, enriched environment with other animals, with free access to food and water, in order to minimize any discomfort and suffering; it was sacrificed for the study of cerebral connectivity). An autoptic tissue sample of the human cerebellum belonging to a woman, 70 years old, was kindly provided by Department of Clinical and Biological Sciences, University of Turin, Italy (sample anonymized for analysis, from a tissue bank; procedure approved by the local Ethical Committee - San Luigi Hospital, Orbassano, Italy, n. 191/INT). Three APPPS1 mice (12 months old, raised in the animal house of NICO research center; authorization Italian Ministry of Health, 17 october 2011) were used as a model of chronic damage (chronic amyloid deposition; Radde et al., 2006); we examined three age matched C57BL/6 as wild-type littermates (authorization as for the APPPS1 mice).

Animals were deeply anesthetized (ketamine 100 mg/kg - Ketavet, Bayern, Leverkusen, Germany - and xylazine 33 mg/kg body weight (5 mg/kg in mice) - Rompun; Bayer, Milan, Italy - for rabbit and guinea pig; sodium pentobarbital 50 mg/kg, i.p., for cat; ketamine 5–10 mg/kg, i.m. and metomidine 30 mg/kg, i.m., for monkey) and perfused intracardially (apart from sheep, perfused in the head through the carotid artery after slaughtery) with a heparinized saline solution followed by 4% paraformaldehyde in 0.1 M sodium phosphate buffer, pH 7.4. Brains were then postfixed (2 hs, mice; 6 hs, rabbits and guinea pig; overnight, monkey; 48 hs, sheep), cryoprotected with increasing concentration of sucrose/0.1 M PB, till 30%, frozen at−80°C, and cryostat sectioned (40 µm thick). The human cerebellar tissue was fixed in formalin for 1 hr then in 4% paraformaldehyde in 0.1 M sodium phosphate buffer, pH 7.4, overnight.

### BrdU injections and surgical procedures

Nine mice received one daily injection of BrdU (Sigma-Aldrich; 40 mg/Kg) for 5 consecutive days and then were killed 5 days (n = 3), 10 days (n = 3), 30 days (n = 3), after the last injection. Six rabbits received one daily injection of BrdU for 5 consecutive days and then were killed 5 days (n = 2, peripuberal), 10 days (n = 4; 2 peripuberal, 2 young), after the last injection. The cat received one daily injection of BrdU for 2 consecutive days and was killed 19 days after the last injection.

Stab wound was performed on three 3 months old CD1 mice. Surgical procedures were carried out under deep anesthesia (ketamine, 100 mg/Kg; Ketavet, Bayern, Leverkusen, Germany; xylazine, 5 mg/Kg; Rompun; Bayer, Milan, Italy). A stab-wound in the right cerebral cortex (Bregma from−0.4 mm to−2 mm, laterolateral 1.5 to 2.5 mm) encompassing both gray and white matter was performed, then animals were killed 15 days after lesion. The controlateral part of the cortex was used as control.

### Immunohistochemistry

Immunohistochemical reactions were performed on free-floating sections incubated in blocking buffer (5% normal serum, 0.3% Triton X-100 in 0.01 M PBS, pH 7.4) for 1 h at RT, and then incubated for 24–48 h at 4°C in a solution of 0.01 M PBS, pH 7.4, containing 0.1–1% Triton X-100, 2% normal serum and the primary antibodies (Table 2). For BrdU staining, DNA was denatured in 2N HCl, 0.5% Triton X-100 for 30 min at 37°C. Sections were then rinsed in 0.1 M borate buffer, pH 8.5 for 20 minutes. Following primary antisera incubation, sections were incubated with appropriate solutions of secondary AMCA-conjugated (1∶200; Jackson ImmunoResearch, West Grove, PA), cyanine 3 (Cy3)-conjugated (1∶800; Jackson ImmunoResearch, West Grove, PA), Alexa488-conjugated (1∶400; Molecular Probes, Eugene, OR) and dylight 649-conjugated (1∶400, Jackson ImmunoResearch, West Grove, PA) antibodies, for 2 hours RT. GD/17 was detected with the high sensitivity Tyramide signal amplification system (Perkin Elmer Life Sciences). Sections were counterstained with 4’,6-diamidino-2-phenylindole (DAPI, KPL, Gaithersburg, Maryland USA), mounted with MOWIOL 4-88 (Calbiochem, Lajolla, CA) and examined using an E-800 Nikon microscope (Nikon, Melville, NY) connected to a colour CCD Camera, and a Leica TCS SP5 (Leica Microsystems, Wetzlar, Germany) confocal microscope.

### Image processing and data analysis

All images were collected with the confocal microscope. Images were processed using Adobe Photoshop CS4 (Adobe Systems, San Jose, CA). Only general adjustments to color, contrast, and brightness were made.

Quantitative analysis (cell densities, marker coexpression, soma diameters and cell process lenght) were performed by means of the Neurolucida software (Micro-Brightfield, Colchester,VT), Imaris 7.4.2 software (Bitplane AG, Zurich, Switzerland) or by confocal analysis. Countings has been conducted on 3 to 5 representative coronal (brain) or sagittal (cerebellum) cryostat sections, from each animal. Three animals were analyzed for each time point or experimental condition. For cell densities: the areas of the regions of interest were determined per section using a suitably calibrated Neurolucida-microscope setup. Data are expressed as densities (cell/mm^2^) and presented as means ± SEM. For multiple/double staining, the number of inspected cells ranged from 50 to 300 in total / 3 animals (see also S2). For Ki67 and BrdU staining, at least 50 cells in total / 3 animals were analyzed (see also S2). For morphometric analysis (soma diameters, total process lenght), at least 30 cells were randomly chosen in each area analyzed in three animals. We analyzed three different CNS areas: cerebral cortex, corpus callosum and cerebellar cortex. The soma diameter was calculated for each cell by measuring its minimum (min) and maximum extent (max) in two orthogonal directions (Fig. S2B). Average soma diameter was obtained averaging the two values. The total length of mMap5 cell processes was calculated on confocal images processed with Imaris 7.4.2 software, using the “Autopath” filament function, to trace labeled processes. All morphometric data were presented as means± SEM.

Statistical analysis was carried out by the Statistical Package for the Social Science 14.0 (SPSS, Chicago, IL) and included two way ANOVA test followed by Bonferroni's post hoc analysis (to compare mean values) and Student's t test (to compare two groups). P<0.05 was considered statistically significant.

### Western blots

Animals were sacrificed at 40 days and 3 months (mouse, n = 6), 3,5 months and 1 year (rabbit, n = 6); brains were collected and then immediately homogenized in a glass-Teflon Potter homogenizer in an ice-cold lysis buffer containing 20 mM Hepes, pH 7.5/10 mM KCl/1.5 mM MgCl2/1 mM ethylenediaminetetraacetic acid (EDTA)/1 mM ethylene glycol tetraacetic acid (EGTA)/1 mM DTT/0.5% CHAPS/complete protease inhibitors; Roche Cat. No. 11 697 498 001). The homogenates were centrifuged at 12000 rpm for 15 min at 4°C. Protein concentration was determined using a Bradford assay #23236. Proteins extracts (50 µg) were separated on SDS-PAGE (6% polyacrilamide) and transferred to polyvinylidene difluoride membranes. Then the membranes were blocked in 5% nonfat milk in tris buffered saline (TBS)-T (200 mM Tris and 1.5 M NaCl with 0.1% Tween 20) and were incubated with primary antibody diluted in TBS-T overnight at 4°C. The membranes were washed and incubated with secondary horseradish peroxidase-coupled antibodies (Santa Cruz Biotechnology, Santa Cruz, CA) in TBS-T for 1 hour at room temperature. After the final washes, the proteins were detected by enhanced chemiluminescence. The bands were quantified using Quantity One® 1-D Analysis Software (Bio-Rad Laboratories) and values were normalized with respect to Vinculin. The values were expressed as a percentage relative to the sham level of OD. The values of the old animals were expressed relatively those of the young animals of the same species. The antibodies and dilution used were as follows: anti-Map5 polyclonal 1∶1000 (see Table 2), anti-vinculin, poyclonal goat, 1∶1000 (Santa Cruz).

## Supporting Information

Figure S1 **Staining with anti-Map5 antibodies used in this study on the olfactory bulb of rabbits and mice.** Both mouse monoclonal and goat policlonal anti-Map5 reveal the same pattern of immunocytochemical distribution in the olfactory bulb (OB) of different mammalian species. As previously described [11], Map5 (Map1B) is heavily expressed in the olfactory nerve layer (ONL) and external plexiform layer (EPL) of young/adult animals. GL, glomerular layer; GrL, granule layer. Scale bars: 50 µm.
(TIF)

Figure S2 **Quantifications carried out on cell processes of mMap5 cells in rabbit and mouse, and on cell bodies of mMap5 and Ng2+cells in mouse.** A, The entire extension of mMap5 cell processes was drawn using Imaris software (left) in grey and white matter regions of rabbit and mouse. Results (right) indicated that mMap5 cells have longer cell processes (cell process total length) in rabbit than in mouse in both the grey matter regions analyzed (cerebral cortex, Cx; cerebellar cortex, Crb), whereas no significant differences were detectable in white matter (corpus callosum, CC). B, the soma diameter was calculated for each cell by measuring its minimum (min) and maximum (max) extent in two orthogonal directions (middle) and averaging the two values (right). Soma diameters of Ng2+cells are prevalently elongated and rather constant in all regions, whereas those belonging to mMap5 cells are prevalently round-shaped in grey matter regions and elongated in white matter. On the whole, the mMap5 cell somata are more heterogeneous. As expected, the average soma diameters are not significantly different.
(TIF)

Figure S3 **Tables with raw data used for quantifications of newly generated cells and subpopulations of mMap5 expressing different markers.**
(DOCX)

Figure S4 A. Map5/β-Tub (Tuj1) double staining in the cerebellum of rabbit and mouse. No overlapping between the two antigens is detectable. B, High magnification confocal images of Map5 staining in the SVZ. Note that many ependymal cells (e) are stained with the anti-Map5 antibody; the Map5 staining is not overlapping with GFAP, and partially overlapping with DCX. LV, lateral ventricle; dlc, dorso-lateral corner; vlw, ventral-lateral wall.
(TIF)
