# Supplementary figures and images for: Cellular and Molecular Characterization of Multipolar Map5-Expressing Cells: A Subset of Newly Generated, Stage-Specific Parenchymal Cells in the Mammalian Central Nervous System
Source: PLoS One. 2013 May 7;8(5):e63258. doi: 10.1371/journal.pone.0063258 (PMC3647045; doi:10.1371/journal.pone.0063258)

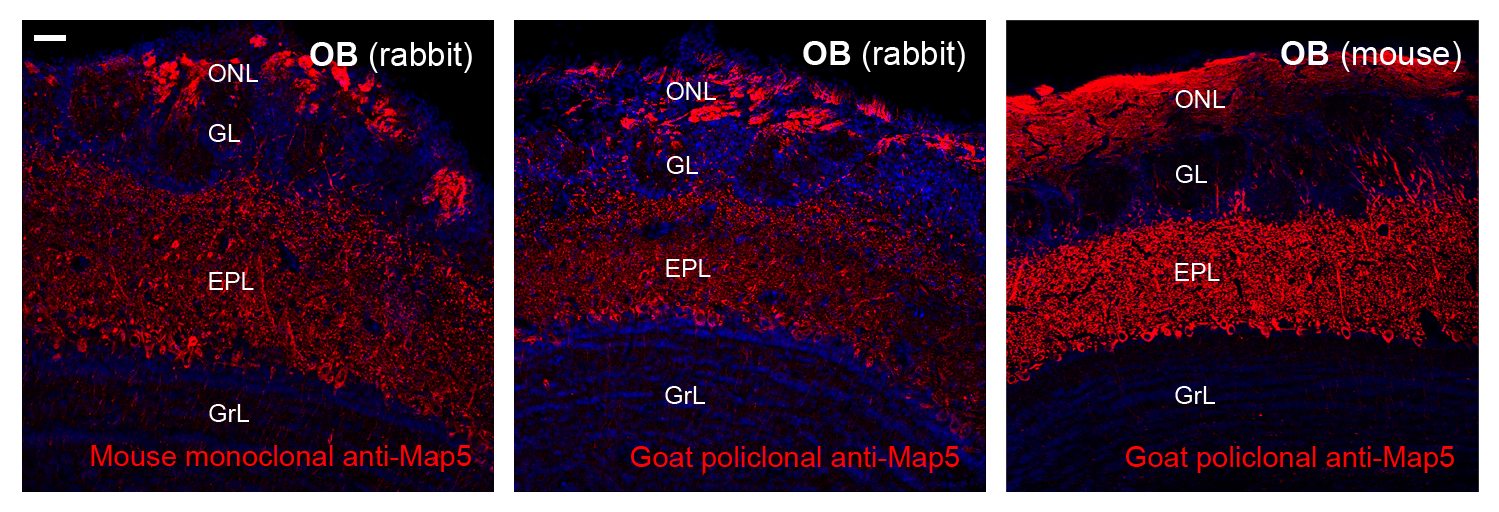

Supplement: Figure S1 — Staining with anti-Map5 antibodies used in this study on the olfactory bulb of rabbits and mice. Both mouse monoclonal and goat policlonal anti-Map5 reveal the same pattern of immunocytochemical distribution in the olfactory bulb (OB) of different mammalian species. As previously described [11], Map5 (Map1B) is heavily expressed in the olfactory nerve layer (ONL) and external plexiform layer (EPL) of young/adult animals. GL, glomerular layer; GrL, granule layer. Scale bars: 50 µm. (TIF) [file pone.0063258.s001.tif]

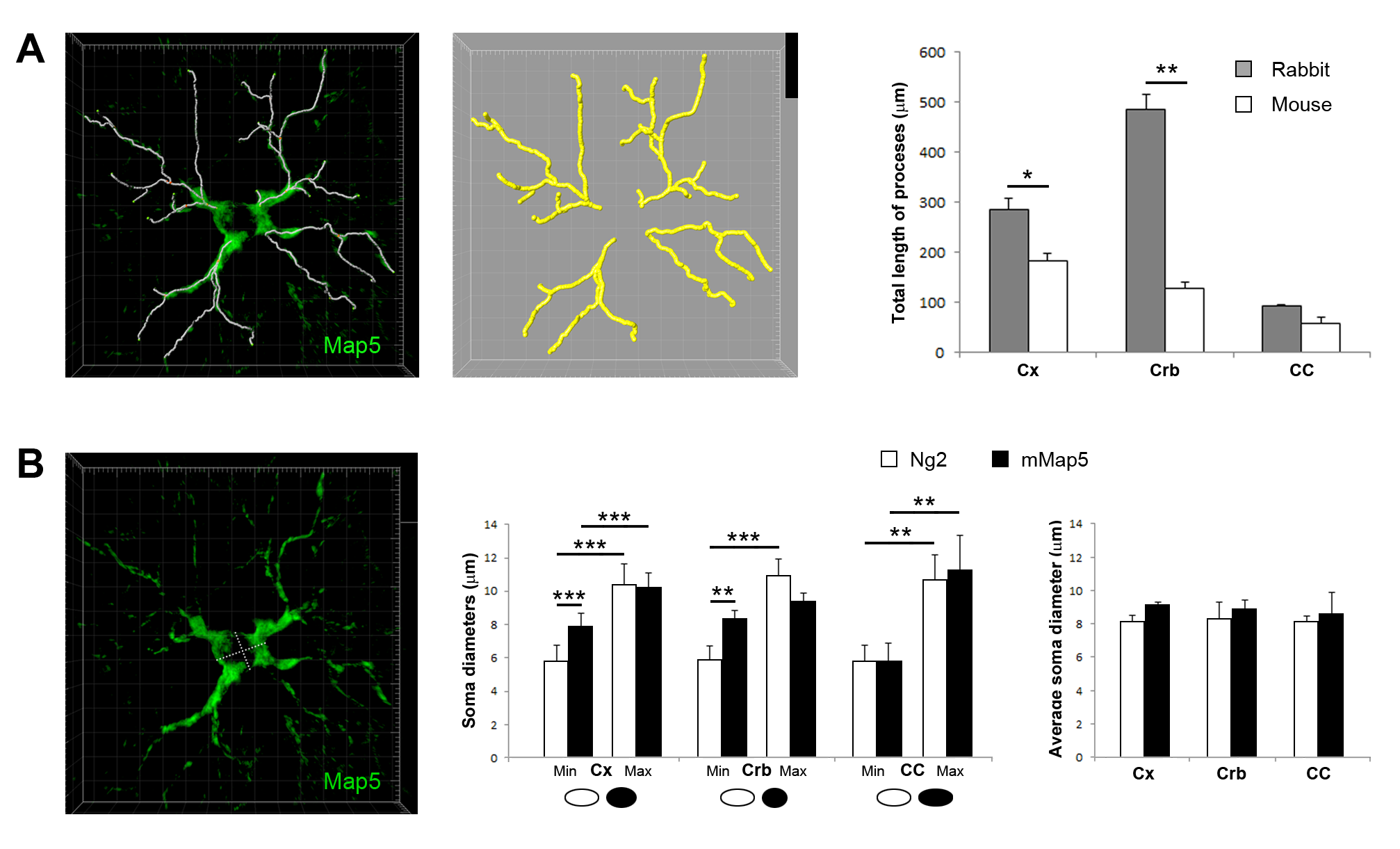

Supplement: Figure S2 — Quantifications carried out on cell processes of mMap5 cells in rabbit and mouse, and on cell bodies of mMap5 and Ng2+cells in mouse. A, The entire extension of mMap5 cell processes was drawn using Imaris software (left) in grey and white matter regions of rabbit and mouse. Results (right) indicated that mMap5 cells have longer cell processes (cell process total length) in rabbit than in mouse in both the grey matter regions analyzed (cerebral cortex, Cx; cerebellar cortex, Crb), whereas no significant differences were detectable in white matter (corpus callosum, CC). B, the soma diameter was calculated for each cell by measuring its minimum (min) and maximum (max) extent in two orthogonal directions (middle) and averaging the two values (right). Soma diameters of Ng2+cells are prevalently elongated and rather constant in all regions, whereas those belonging to mMap5 cells are prevalently round-shaped in grey matter regions and elongated in white matter. On the whole, the mMap5 cell somata are more heterogeneous. As expected, the average soma diameters are not significantly different. (TIF) [file pone.0063258.s002.tif]

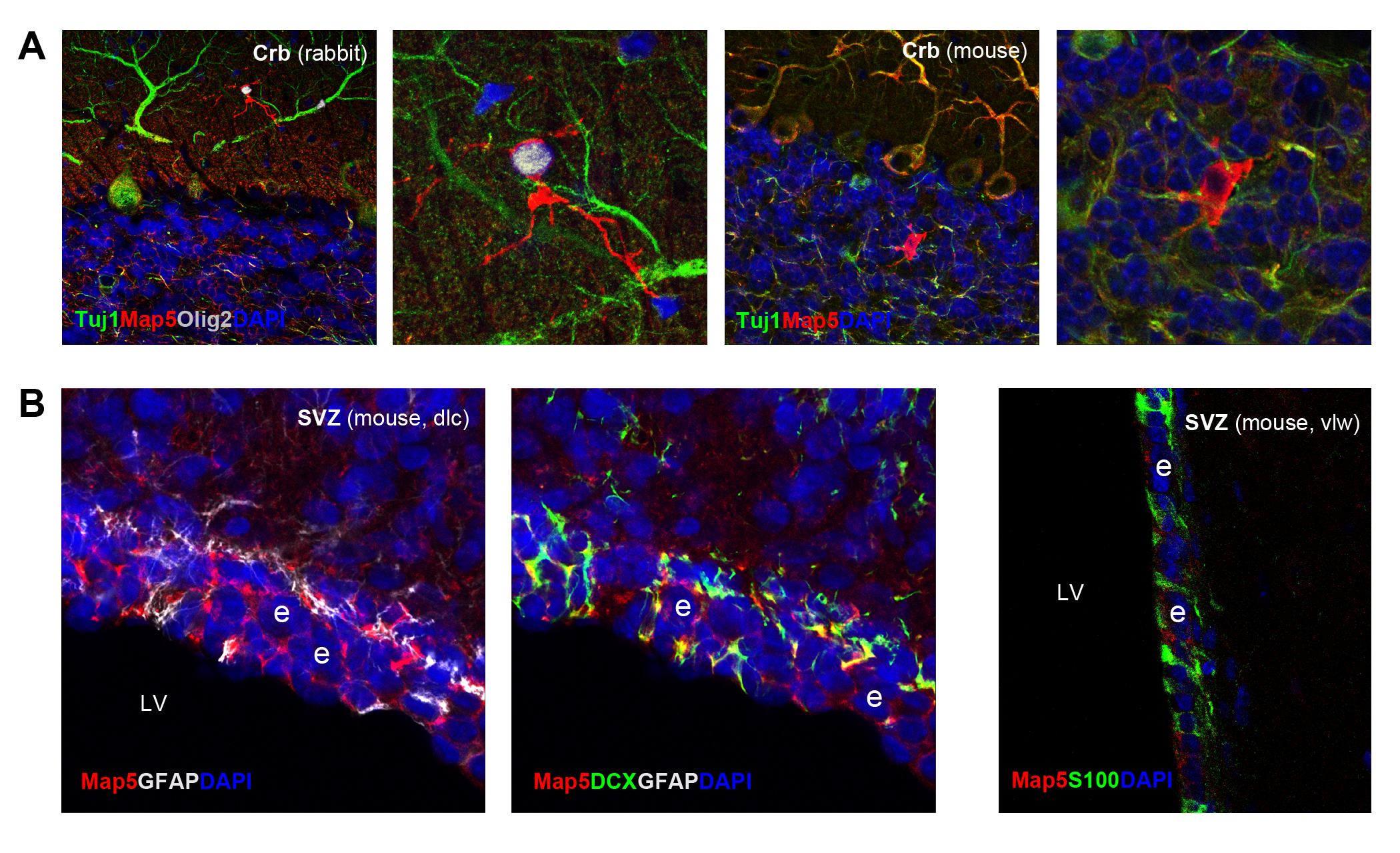

Supplement: Figure S4 — A. Map5/β-Tub (Tuj1) double staining in the cerebellum of rabbit and mouse. No overlapping between the two antigens is detectable. B, High magnification confocal images of Map5 staining in the SVZ. Note that many ependymal cells (e) are stained with the anti-Map5 antibody; the Map5 staining is not overlapping with GFAP, and partially overlapping with DCX. LV, lateral ventricle; dlc, dorso-lateral corner; vlw, ventral-lateral wall. (TIF) [file pone.0063258.s004.tif]
