# Supplementary material for: Cellular and Molecular Characterization of Multipolar Map5-Expressing Cells: A Subset of Newly Generated, Stage-Specific Parenchymal Cells in the Mammalian Central Nervous System
Source: PLoS One. 2013 May 7;8(5):e63258. doi: 10.1371/journal.pone.0063258 (PMC3647045; doi:10.1371/journal.pone.0063258)
Supplement: Figure S3 — Tables with raw data used for quantifications of newly generated cells and subpopulations of mMap5 expressing different markers. (DOCX) [file pone.0063258.s003.docx]

**A**

|  | | **Ng2** | **GPR17** | **Map5** | **GST-π** |
| --- | --- | --- | --- | --- | --- |
| **Ki67** | | most | rare | none | none |
| **BrdU** (5+5 days) | Cx  Cc | 100 out of 120  60 out of 70 | 56 out of 133  38 out of 130 | 2 out of 213  5 out of 111 | 0 out of 102  0 out of 97 |
| **BrdU** (5+10 days) | Cx  Cc | 37 out of 51  40 out of 70 | 53 out of 130  25 out of 71 | 1 out of 89  14 out of 70 | 2 out of 92  0 out of 52 |
| **BrdU** (5+30 days) | Cx  Cc | 42 out of 58  22 out of 68 | 8 out of 179  5 out of 122 | 1 out of 104  6 out of 97 | 8 out of 66  9 out of 50 |

Number of newly generated cells expressing different antigens and revealed with endogenous (Ki67) and exogenously administered (BrdU) cell proliferation markers. Values indicate the number of double-stained cells out of all Ki67/BrdU+ nuclei (expressed in percentage in Fig. 4), counted in the cerebral cortex (Cx) and corpus callosum (Cc). Mice (n=3)

**B**

|  | | **GPR17** | **Sox2** | **Sox9** | **Olig2** | **Sox10** |
| --- | --- | --- | --- | --- | --- | --- |
| **mMap5** | **Cx** | ND | 47 out of 66 | 26 out of 181 | 140 out of 149 | 74 out of 76 |
|  | **Cc** | ND | 42 out of 94 | 18 out of 129 | 120 out of 128 | 83 out of 86 |
|  | **Crb (g)** | 55 out of 98 | 130 out of 257 | 139 out of 300 | 143 out of 155 | 116 out of 119 |
|  | **Crb (w)** | 42 out of 50 | 35 out of 87 | 50 out of 82 | 60 out of 66 | 57 out of 63 |

Quantitative evaluation of the subpopulations of mMap5 expressing different markers in different brain regions (expressed in percentage in Fig. 3). ND: not determined. Rabbits (n=3)
